# Computational epitope heterogeneity analysis in immunostainings from antibody-dilution series

**DOI:** 10.1038/s42003-026-09517-x

**Published:** 2026-02-02

**Authors:** Dominik Tschimmel, Momina Saeed, Maria Milani, Steffen Waldherr, Tim Hucho

**Affiliations:** 1 Translational Pain Research, Department of Anesthesiology and Intensive Care Medicine, Faculty of Medicine and University Hospital Cologne, University of Cologne, Cologne, Germany; 2 Department of Functional and Evolutionary Ecology, University of Vienna, Vienna, Austria

**Keywords:** Fluorescence imaging, Single-cell imaging

## Abstract

Antibodies are widely used in life sciences and medical therapy. Broadly applicable methods to determine epitope heterogeneity in immunostaining systems are missing. Here, we present a simple-to-use approach to characterize and quantify antibody binding properties that constitute the staining directly in the system of choice. We determine an epitope heterogeneity on the basis of a computational analysis of antibody-dilution immunofluorescence stainings. This allows us to choose signal-specificity maximizing dilutions and to improve signal quantification. Furthermore, the computational analysis provides approaches to obtain a single-channel antibody multiplexing. Our approach could help improving immunostainings in many laboratories by guiding the choice of antibody dilution, by increasing the possibility of antibody-multiplexing in the same color-channel and by allowing for the analysis of binding targets of multi-specific antibodies.

## Abstract (web summary)

Computational epitope heterogeneity analysis from simple dilution-series experiments can improve immunostaining in many laboratories. The analysis enables a computational single-color channel multiplexing based on binding properties.

## Introduction

Antibody-based applications range from basic research and diagnostics^1,2^ to therapeutic intervention^3–5^. Accordingly, the purpose varies from, e.g., target identification and quantification to the delivery of pharmaceuticals. Central for each of these applications are target accessibility and binding specificity. Nevertheless, information about these parameters is rarely available, and in even fewer cases, these parameters have been obtained for the exact system of choice.

Like any other protein-protein interaction, antibodies bind to target proteins by biochemical, non-covalent interactions. Thus, the strength of the interaction depends on the primary, secondary, tertiary, and quaternary structure of the involved proteins as well as factors such as binding-site accessibility, pH, ionic strength, temperature, and exposure time^6^. This creates heterogeneous binding environments that depend on the subcellular domains of the epitopes. Heterogeneous binding can even occur for a single epitope in a fully purified system^7^. Broadly applicable methods to assess the heterogeneity of antibody binding in complex systems are missing. The current default approach, surface plasmon resonance, is best suited for the analysis of at least in part purified protein systems, relying on strongly altered binding environments^8^.

This can result in a misguided choice of antibody concentrations, an undefined ratio of specific versus unspecific binding, misleading interpretation, and inaccurate quantification. Furthermore, it misses the opportunity to analyze function-defining changes of the protein environment.

We propose a computational analysis of antibody-dilution-series-derived immunostainings to characterize the epitope accessibilities and the target-heterogeneity. The underlying model describes antibody accumulation as a function of antibody concentration and binding constants, comprising the raw chemical antibody-epitope interaction as well as system-specific effects. The top-down description of the computational analysis can be summarized as a curve fitting of dilution-series data to determine the unknown binding constants. Since the binding constants comprise system-specific effects, contrary to common modeling approaches that aim to model out these effects^9,10^, they characterize the antibody-binding behavior of the biological system under investigation. Since diffusion limitations and binding obstructions are central components of the binding behavior, we call the binding constants “accessibility constants”.

In detail, our computational analysis yields an accessibility histogram, which describes the distribution of epitopes in the biological system with respect to their accessibility constants. This allows the identification of distinct groups of epitopes that exhibit a similar binding behavior. As such, the distinction does not correspond directly to biological epitope types. Identical epitopes can behave completely differently, based on their location within the cell. Likewise, biologically different epitopes can have similar binding behaviors. To avoid confusion, we use the technical name “epitope class” for the accessibility classification.

In this paper, we show that immunostaining violates the equilibrium assumption of isotherms, which are commonly used to describe the dose-response relationship of protein binding^11,12^. In doing so, we validate our proposed accumulation model. After establishing the accumulation model, we demonstrate that the resulting accessibility histograms characterize the binding behavior of antibodies for common immunostaining protocols. In particular, we demonstrate how the accessibility histogram can be used to detect potentially unspecific staining and to select antibody concentrations that optimize the ratio between specific and unspecific binding. For this purpose, we created a validation system consisting of HeLa cells and exemplary antibodies directed against NF200 and the ribosomal protein RPS11. To corroborate the connection between the accessibility histogram and the binding behavior of antibodies further, we investigate the conformation-sensitive epitope of the regulatory subunit RII beta of PKA^13^. In that way, we show that conformational changes are reflected in accessibility histograms. Finally, we propose a multi-staining protocol that uses the information of the accessibility histogram to achieve a binding-property-guided, computational multiplexing of a single color channel.

## Results

### Antibody accumulation and accessibility histograms

To describe protein and antibody binding, mass action kinetics, or Langmuir kinetics (mathematically identical up to units), are commonly used^11,12,14^. The resulting rate equation relates the concentration of bound antibodies *x*(*t*), the concentration of free antibodies *a*(*t*), the concentration of total epitopes *g*, and two rate constants for binding *k*_*a*_ and unbinding *k*_*d*_:

$$\frac{d}{dt}x(t)={k}_{a}a(t)(g-x(t))-{k}_{d}x(t).$$

Assuming an equilibrium state yields the well-known Langmuir isotherm

$${x}_{eq}=\frac{g}{1+\frac{{k}_{d}}{{k}_{a}{a}_{eq}}}.$$

However, the equilibrium assumption fails for common immunostaining protocols that contain several washing steps between antibody incubation and microscopy. Consequently, only antibodies that withstood washing can be observed, such that *k*_*d*_ ≈ 0 must be assumed for microscopy results,

$$\Rightarrow \,\frac{d}{dt}x(t)={k}_{a}a(t)(g-x(t)).$$

When there is more than one epitope class, the equation becomes

$$\frac{d}{dt}{x}_{i}(t)={k}_{a,i}a(t)({g}_{i}-{x}_{i}(t))\,\,\forall \,i\in \{1,\ldots ,N\}.$$

This system has no analytical solution for the depletion case $a(t)=a-\beta {\sum }_{j=1}^{N}{x}_{j}(t)$ that describes antibody incubation in a well plate or Petri dish with finite volume.

**Fig. 1:**
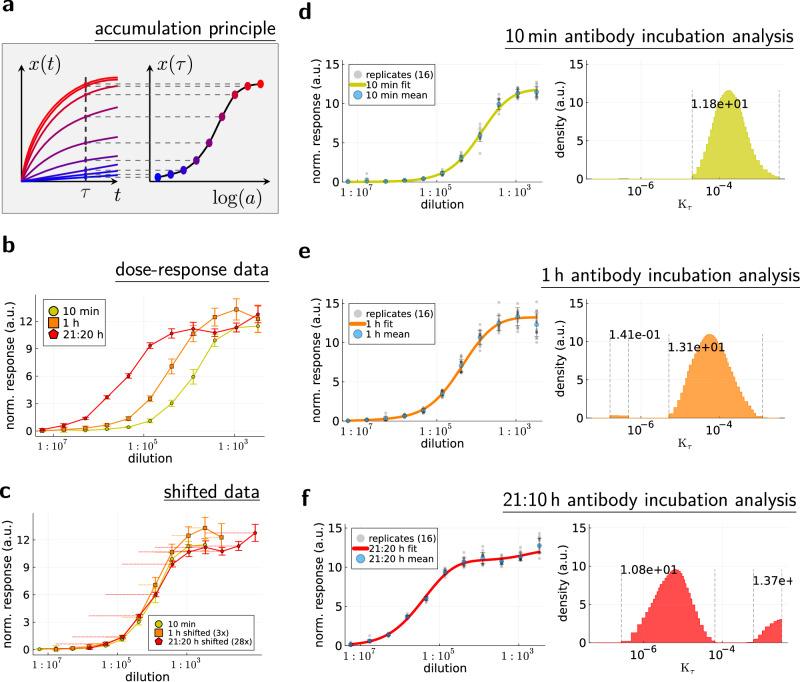
Validation of the accumulation model with incubation time experiments. **a** Illustration of concentration-dependent binding rates that produce dose-response curves when all conditions are incubated for the same time *τ*. **b** Dose-response curves (mean values of *n* = 16 replicates; error bars = sample std) of an anti-NF200 antibody in HeLa cells for three different antibody incubation times (10 min,1 h, 21:20 h). **c** Same dose-response curves as (**b**) but plotted for antibody dilutions multiplied with a constant factor (3x and 28x, respectively), which becomes a shift in a logarithmic scale. The similarity of the dose-response curves indicates that increasing the incubation time has a similar (but not identical) effect to increasing the antibody concentration. **d–f** Accessibility histograms for the dose-response curves (mean values of *n* = 16 replicates; error bars = sample std). Annotations in the accessibility histograms indicate the amount of equivalent epitopes (i.e., epitope concentration measured in the arbitrary unit “normalized response”) contained in the respective intervals enclosed by dashed lines.

Using the model with antibody depletion for parameter estimation would require numerical solutions and additional mathematical considerations that go beyond the scope of this paper (additional mathematical considerations can be found in the preprint https://arxiv.org/abs/2409.06895). Instead of using numerical solutions, we provide a worst-case data correction as method to detect depletion effects that cannot be neglected. Applying this worst-case correction to our data showed that our results do not change. The only noteworthy difference can be observed for the 21:20 h incubation (Fig. 1f), which actually corroborates the accumulation assumption. See Supplementary Note 4 for further information.

Assuming *a*(*t*) = *a*, contrary to the experimental setting, and *x*(0) = 0 yields

$${x}_{i}(t)={g}_{i}(1-{e}^{-{k}_{a,i}at})\,\,\forall \,i\in \{1,\ldots ,N\}\,,$$

which can be treated as an empirical model. Consequently, the rate constants ${\{{k}_{a,i}\}}_{i=1}^{N}$ become abstract, system-characterizing parameters that encompass multiple effects, including the behavior of the “washed-away antibodies” and the behavior of secondary antibodies.

Note that modeling secondary antibodies would become impractical for many applications. At least as long as secondary antibodies are used to make primary antibodies detectable. Equations that model the binding of secondary antibodies would only introduce additional model parameters. These secondary-antibody parameters could not be determined separately from the primary-antibody parameters with experiments in which only the primary-antibody concentration is varied. The resulting experimental data would only contain the combined effect of primary-antibody + secondary-antibody binding. Yet, simultaneously varying the primary-antibody concentration together with the secondary-antibody concentration would require significantly more experimental conditions, contradicting our goal to provide an easily applicable analysis tool.

In consequence, good experimental practices should be followed by using the same concentration of secondary antibodies for each condition, such that the primary-antibody concentration is the only independent variable. In this way, the model describes the properties of the actual signal that is observed in the end: the combination of primary and secondary antibodies.

Following good experimental practices also means that all conditions of a dilution series should be incubated for roughly the same time *τ*. Thus, the dose-response relationship is given by

1 $$x(a)={\sum }_{i=1}^{N}{g}_{i}(1-{e}^{-{k}_{a,i}a\tau }):={\sum }_{i=1}^{N}{g}_{i}(1-{e}^{-\frac{a}{{K}_{\tau ,i}}}),$$

where we defined the “accessibility constants” ${K}_{\tau ,i}:=\frac{1}{{k}_{a,i}\tau }$, which have the same dimension as the antibody concentration *a*. This name is motivated by the fact that diffusion obstructions constitute a major effect that distinguishes the experimentally observable binding behavior in complex systems from the raw chemical binding properties of purified compounds.

In the accumulation model, the experimentally observed dose-response relationship arises from the antibody-concentration-dependent accumulation rates $\frac{{K}_{\tau ,i}}{a}$, as illustrated in Fig. 1a. Because of the product *aτ* in Eq. (1), doubling the incubation time *τ* should have the same effect as doubling the antibody concentration *a* (which can be observed as a shift in a logarithmic plot). Simple dilution series experiments for different antibody incubation times partially confirm the postulated accumulation principle (Fig. 1b, c). However, the relationship is not linear, as expected by the term *aτ*. Instead, increasing the incubation time has a weaker effect than increasing the concentration, and the larger the difference in the effect size.

The reason for this discrepancy is probably the undetectable antibody behavior prior to the washing steps. The accumulation model only describes detectable antibodies that withstand washings. Yet, antibodies may still bind temporarily before they are washed away in the end. Increasing the antibody concentration effectively blocks more temporary binding spots, allowing for higher levels of permanently bound antibodies compared to increasing the incubation time. Temporary binding would also explain why increasing the incubation time also increases the level of bound antibodies. Essentially, antibodies get stuck for a certain amount of time before they become unbound and get another chance to bind permanently.

For parameter estimation, the potentially large and unknown number of epitope classes is inconvenient and requires additional selection criteria. One approach is to approximate Eq. (1) as Fredholm integral equation

2 $$x(a)=\int _{0}^{\infty }g({K}_{\tau })(1-{e}^{-\frac{a}{{K}_{\tau }}})\,d{K}_{\tau }\,,$$

where *g*(*K*_*τ*_) describes the distribution of epitopes with respect to the accessibility constant (cf. ^7^). We solve this equation numerically by approximating *g*(*K*_*τ*_) as Riemann sums with iteratively refined intervals similar to^15,16^. Since a dilution series constitutes a sparse data set for the dose-response relationship *x*(*a*), the resulting numerical optimization problem is ill-defined and requires additional regularization^17^. We implemented regularization as prior probability in the Bayesian framework (cf. ^16,18^), using a smoothness regularization for all analyses in this paper. The resulting Riemann sum approximation is then normalized so that the visual area under the graph, if plotted logarithmically, corresponds 1:1 to the respective epitope concentration, which corresponds 1:1 to the effect on the dose-response curve (see "Methods" and Supplementary Note 2.2). Because we called *K*_*τ*_ accessibility constant, we call the resulting plot “accessibility histogram”.

Choosing an empirical model results in accessibility histograms that do not depend on the choice of units, as proven in Supplementary Note 3. Thus, antibody concentrations can be measured as dilution quotients $(1:n\,\widehat{=}\,\frac{1}{n})$ from a stock dilution and the response value can be measured as an arbitrary signal strength. The same arbitrary signal strength unit should also be used for the epitope concentration *g*. Both *x*(*a*) and *g* essentially describe epitopes, where *x*(*a*) considers only the subset of epitopes to which antibodies have bound. To avoid confusion with the dimensionless unit of (particle) numbers, we will use the term “equivalent epitopes” when we speak about the amounts of epitopes measured in arbitrary units. Also note that we use the notation 1:*n* to indicate that concentrations are expressed in terms of dilution quotients. This should not be confused with dilution ratios ("1 part to n parts”), which are not proportional to the actual concentration if used as numbers 1/*n*.

Figure 1d–f shows the accessibility histograms for the dose-response in Fig. 1b. As the dose-response curve moves to the left when the incubation time is increased, so does the major peak in the histogram. This is because the position of *K*_*τ*_-peaks is connected to the antibody concentration of dose-response behavior by $\frac{a}{{K}_{\tau }}$. Hence, the amount of equivalent epitopes that correspond to the peak remains effectively unchanged as well.

However, the peaks are not identical, which becomes more noticeable for a smaller smoothness regularization parameter (Supplementary Fig. 1) and for the worst-case depletion correction (Supplementary Note 4). The major peak disperses into two peaks for *τ* = 21: 20 h, indicating that the incubation time *τ* and the antibody concentration *a* are not perfectly equivalent. This could be explained with the unobserved antibody trajectories as follows: By increasing the incubation time, the chances that an antibody eventually binds are increased. However, since antibodies encounter accessible epitopes (low *K*_*τ*_) along the way to less accessible epitopes, most antibodies bind to accessible epitopes first. This leads to a major difference between accessible and less accessible epitopes, producing two distinct peaks. For increased antibody concentrations, on the other hand, the accessible epitopes get blocked by bound antibodies, allowing the remaining antibodies to reach less accessible epitopes.

Using a different regularization parameter revealed an aspect of the accessibility histogram that one needs to be cautious about. The width of peaks depends on the chosen regularization. In consequence, the width of peaks should not be overinterpreted, and one should focus more on the number and *K*_*τ*_ values of the peaks (cf. ^19^). As a general rule of thumb (feature parsimony), the smoothness regularization parameter should be chosen as large as possible such that the resulting model curve still describes the data points reasonably well. In addition, it is also recommended to consider weaker regularization parameters. For example, since a slight improvement of the model curve can be observed for the incubation time experiments (Supplementary Fig. 1), reducing the smoothness regularization parameter can be justified. Additional information about the regularization parameter can be found in the “Methods” section and in the Supplementary Notes 6 and 8.

### Accessibility histograms describe binding properties

To validate the correspondence between accessibility histograms and antibody binding properties, experimental immunocytochemical data with an antibody that detects two clearly separable epitopes would be best. We created a fully controlled artificial two-epitope-binding system by mixing two monoclonal antibodies directed against two completely different epitopes; we arbitrarily chose NF200 and RPS11. As both antibodies were derived from different hosts, the contribution of each antibody to the total signal intensity (sum of signals) could easily be determined. Furthermore, the relative binding rate constants of the antibodies could be changed by changing mixing ratio of the antibodies.

**Fig. 2:**
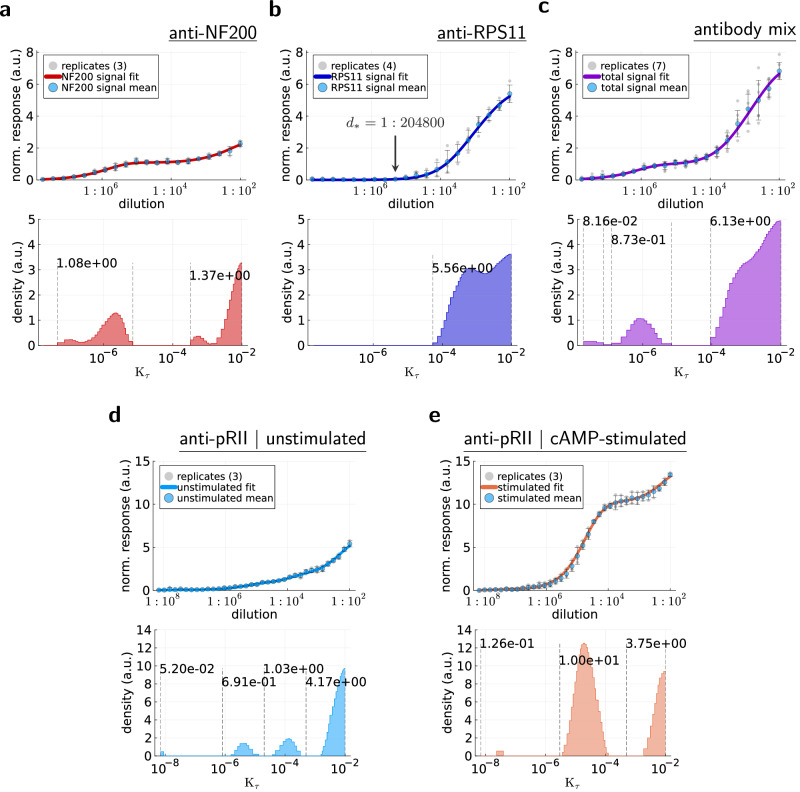
Validation of the accessibility histograms. Dose-response curves and the corresponding accessibility histograms (mean values of replicates (*n* indicated in legends); error bars = sample std). **a** HeLa cells and anti-NF200 antibody. **b** HeLa cells and anti-RPS11 antibody. **c** HeLa cells and anti-NF200-anti-RPS11 mix (total signal). **d** Unstimulated HeLa cells and anti-pRII antibody. **e** cAMP-stimulated HeLa cells and anti-pRII antibody (error bars for lowest dilution quotient set to 0.01, cf. “Methods”). Annotations in the accessibility histograms indicate the amount of equivalent epitopes contained in the corresponding intervals enclosed by dashed lines. The annotation d_*_ = 1: 204800 in (**b**) marks the dilution quotient before the anti-RPS11 signal begins to increase.

Conceptually, the anti-RPS11 antibody was intended to mimic the unspecific signal that is usually most prominent at high antibody concentrations^20^, while the anti-NF200 antibody was intended to mimic the specific signal present at lower antibody concentrations. Accordingly, the anti-NF200 antibody was used at higher concentrations, both when used individually and in the antibody mix (see “Methods”). Figure 2a–c shows the dose-response curves for the individual antibodies and for the total signal of the antibody mix, as well as the corresponding accessibility histograms.

As intended, the anti-RPS11 dose-response behavior mimics an unspecific background signal that appears only at large dilution quotients (Fig. 2b). The anti-NF200 dose-response behavior shows a first signal increase already at small dilution quotients, with a second increase at large dilution quotients, which might be due to actual nonspecific binding (Fig. 2a). For later use, we mark the dilution quotient d_*_ = 1: 204800 before the anti-RPS11 signal begins to increase. Unsurprisingly, the total signal of the antibody mix is a perfect superposition of the individual antibody dose-response behaviors.

A similarly inconspicuous but far more important superposition can be observed in the accessibility histograms. The histogram for the antibody mix contains the individual peaks of the anti-NF200 histogram and the anti-RPS11 histogram (for *K*_*τ*_ ≥ 10^−4^ the peaks combine). This validates that the accessibility histogram describes the dose-response behavior of antibodies. In itself, the descriptive capabilities of the accessibility histogram do not seem to provide much value. However, while it is easy to obtain the superposition of the individual dose-response curves without actual experiments by simply adding the signal values, the opposite direction is less trivial. In a generic dilution series experiment of a single antibody that shows a complex dose-response behavior, it is not clear how many distinct binding types underlie the dose-response curve. That is, knowing only the dose-response curve in Fig. 2c, it is far from trivial to predict the existence and shape of the dose-response curves of the anti-NF200 antibody and the anti-RPS11 antibody. Yet, the existence of two separate peaks in the accessibility histogram (Fig. 2c) makes it obvious that there are at least two epitope classes. Using a weaker regularization (Supplementary Fig. 2) indicates that there might be 3 peaks corresponding to 3 epitope classes.

In a second validation experiment, we tested the behavior of our computational analysis in an example where the accessibility of an epitope is altered. Isensee et al.^13^ showed that the regulatory subunit of inactive PKA type II (PKA-II) contains a constitutively phosphorylated regulatory subunit and that this phospho-epitope (pRII-epitope) is rendered inaccessible (for a pRII-detecting antibody) by the non-covalent interaction with the catalytic subunits in the inactive holoenzyme. Furthermore,^13^ showed that during the course of activation by cAMP, the distance between regulatory and catalytic PKA subunits increases, up to a complete detachment of the fully active catalytic subunit of PKA, rendering the pRII-epitope accessible for the anti-pRII-antibody. As there is always cAMP in any cell, we would expect two histogram peaks for unstimulated cells, one low-*K*_*τ*_ peak corresponding to the accessible pRII-epitope of activated PKA, and a second, high-*K*_*τ*_ peak for the rather closed conformation. Stimulation with cAMP should strongly reduce the high-*K*_*τ*_ peak, resulting in a single, low-*K*_*τ*_ peak in the accessibility histogram.

Figure 2d–e shows anti-pRII dose-response curves and the corresponding accessibility histograms for cAMP-stimulated and unstimulated HeLa cells. At first glance, the accessibility histograms seem to behave as expected. Unstimulated cells show two peaks that could correspond to highly accessible and hardly accessible peaks, together with a third peak at *K*_*τ*_ > 10^−3^ that could be attributed to unspecific staining. However, the two peaks at *K*_*τ*_ < 10^−3^ become a single peak with 5-times more epitopes upon cAMP-stimulation. This suggests that all peaks at *K*_*τ*_ < 10^−3^ correspond to accessible pRII-epitopes. The presence of two peaks for unstimulated cells could then be explained by different cellular locations of PKA with accessible pRII-epitopes. This agrees with the merging of the peaks upon cAMP stimulation, which probably exposes pRII-epitopes all throughout the cell.

**Fig. 3:**
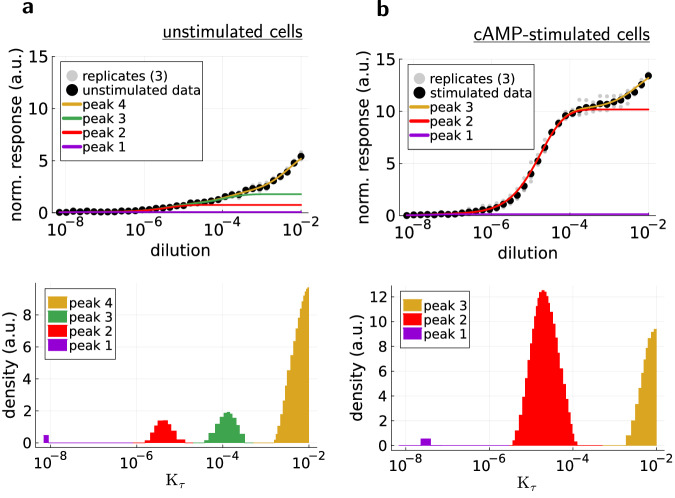
Analysis of individual peaks in the accessibility histogram for the anti-pRII antibody. Contribution of the individual peaks to the dose-response curve (color-matched) of the anti-pRII antibody in unstimulated HeLa cells (**a**) and cAMP-stimulated HeLa cells (**b**). The contributions of the peaks are added on top of each other from left (low *K*_*τ*_) to right (high *K*_*τ*_). Data points are the mean values of *n* = 3 replicates.

The claim that the peak at *K*_*τ*_ > 10^−3^ belongs to unspecific background staining instead of inactive PKA with inaccessible pRII-epitopes can be motivated by the following observations. First, this peak is almost the same for cAMP-stimulated and unstimulated cells. Second, it does not cause the signal intensity increase observed for cAMP-stimulation. To see that this is the case, we must model the individual effects of the peaks to the dose-response curves. Because of the term $\frac{a}{{K}_{\tau }}$ in the accumulation model (2), low-*K*_*τ*_ peaks contribute at lower antibody concentrations. That is, they occur first in the dose-response curve (viewed from left to right). Thus, the contributions of the peaks can be added on top of each other from left (low *K*_*τ*_) to right (high *K*_*τ*_) to obtain the combined dose-response curve (Fig. 3), which confirms that the peak at *K*_*τ*_ > 10^−3^ does not produce the intensity increase of cAMP-stimulation.

In addition to HeLa cells, we performed the anti-pRII experiments also in mouse dorsal root ganglion (DRG) neurons (Supplementary Note 5), leading to similar results.

### Accessibility histograms and optimal staining dilutions

The correspondence between peaks in the accessibility histogram and the antibody concentration at which they affect the dose-response behavior can be used as a simple tool to determine the optimal antibody concentration for immunostainings. At least in the sense that contributions of high-*K*_*τ*_ peaks are minimized. Since staining antibodies are usually designed to bind with high affinity to their target, unspecific background staining is most often present at high staining concentrations^20,21^. So, low-*K*_*τ*_ peaks, corresponding to low antibody concentrations, should correlate with the actual signal, and high-*K*_*τ*_ peaks should correlate with potentially unspecific background staining (as seen for the anti-pRII antibody). The general approach is now to determine the antibody concentration before high-*K*_*τ*_ peaks (if there are any) contribute to the dose-response curve. This can be achieved by modeling the individual contributions of the accessibility histogram peaks, as done for Fig. 3. The splitting points where the individual contributions diverge then mark the desired antibody concentrations. For example, the optimal dilution quotient for the anti-pRII antibody and cAMP-stimulated HeLa cells should be around 1:10^4^ (Fig. 3b), which is 20 times more dilute than the vendor suggestion (1: 500).

**Fig. 4:**
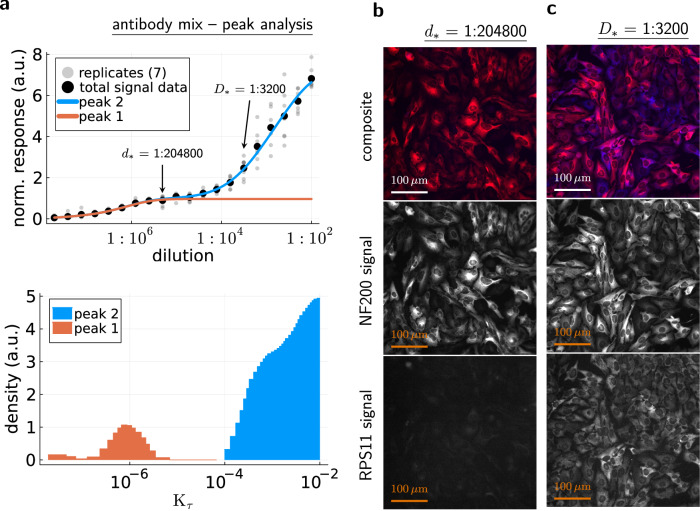
Accessibility histograms can be used to optimize staining concentrations. **a** Individual contributions of the low-*K*_*τ*_ peak (orange, *K*_*τ*_ ≤ 10^−5^) and the high-*K*_*τ*_ peak (azure, *K*_*τ*_ ≥ 10^−4^) to the dose-response curve (total signal data = mean values of *n* = 7 replicates). The splitting point between the individual contributions defines the optimal antibody dilution quotient d_*_. The dilution quotient at which both peaks contribute equally to the dose-response signal is D_*_. **b** Example images for the antibody mix and the dilution quotient d_*_. The brightness of all images was increased for better visibility. On top is the composite image, in the middle is the monochrome image for the anti-NF200 fluorescence signal, and at the bottom is the monochrome image for the anti-RPS11 fluorescence signal. The anti-RPS11 image appears to be a faint copy of the anti-NF200 image because of a 10% channel bleed-through (see Supplementary Fig. 7e). **c** Example images for the dilution quotient D_*_.

Again, the artificial system with the anti-NF200-anti-RPS11 antibody mix serves as a validation system. The unspecific background is mimicked by the anti-RPS11 signal that occurs only at dilution quotients larger than d_*_ = 1:204800 (Fig. 2b). The accessibility histogram yields a similar dilution quotient for the splitting point between the low-*K*_*τ*_ peak contribution (orange in Fig. 4a) and the high-*K*_*τ*_ peak contribution (azure in Fig. 4a). This indicates that accessibility histograms can be used to find optimal staining concentrations, because the total signal of the antibody mix did not contain information about the individual antibody contributions (anti-NF200 and anti-RPS11).

However, there is an important limitation: The accessibility histogram can only provide optimal antibody concentrations for monoclonal antibodies. Polyclonal antibodies, consisting of a mixture of antibodies in an unknown mixing ratio and may exhibit multiple *K*_*τ*_-peaks, because the antibody clones are likely concentrated differently in the mixture. Thus, the correlation argument about signal specificity and staining concentration is no longer valid. Our artificial validation system, consisting of two monoclonal antibodies at different concentrations, demonstrates this phenomenon.

Furthermore, the assumption that yields the optimal concentration relies on the heuristic that unspecific binding occurs at high antibody concentrations. However, the structures that actually produce high-*K*_*τ*_ peaks cannot be inferred from the accessibility histogram alone. This requires additional experiments and analyses. This motivated us to design a method for the visualization of structures that produce high-*K*_*τ*_.

### Computational multiplexing with accessibility histograms

Although the heuristic of signal specificity and staining concentration is not valid for polyclonal antibodies, the ability to control peak contributions by using appropriately chosen antibody concentrations remains unaffected. This provides the opportunity to achieve a binding-property-guided computational multiplexing from a single label.

The general idea for this computational multiplexing is quite simple. First, the accessibility histogram needs to be obtained from dilution series experiments. Then, antibody concentrations are determined at which the peaks reach their maximal contributions before the next peak begins to contribute (from left/low *K*_*τ*_ to right/high *K*_*τ*_). For the last peak (highest *K*_*τ*_), the largest feasible antibody concentration is picked. Finally, the same cell sample is repeatedly stained with the selected antibody concentrations from lowest to highest. Between each staining round, microscopy images of the same region (view field) need to be taken. In this way, the signal increases between the staining rounds correspond to the peaks in the accessibility histogram. These signal increases can be obtained by aligning the images and subtracting them pixel by pixel. For easier interpretation, the difference images can then be combined into a composite image with different colors that correspond to the respective peaks.

As before, our artificial validation system can be used to demonstrate and validate the binding-property-guided computational multiplexing. For the 1st staining round, we chose the dilution quotient *d*_1st_ = 1:102400, which is 2-times more concentrated than the optimal dilution quotient d_*_. This ensures the saturation of the low-*K*_*τ*_ peak (orange, Fig. 4a). Following the multi-staining description above, we would choose 1:100 as the second dilution quotient. However, the anti-NF200 and anti-RPS11 accessibility histogram peaks overlap for *K*_*τ*_ ≥ 10^−4^ (Fig. 2a, b), which complicates the validation. The individual antibody signals could not be used to verify that the signal increase belongs only to the high-*K*_*τ*_ peak (azure, Fig. 4a). Hence, we had to avoid too high dilution quotients for the 2nd staining round to ensure that most of the high-*K*_*τ*_ peak signal consists only of the anti-RPS11 signal. Thus, we picked *d*_2nd_ = 1:6400 for the 2nd staining round.

To demonstrate the general applicability of accessibility histograms, we chose a validation system that uses secondary antibodies (always at the same concentration). For our computational multiplexing, secondary antibodies require additional correction methods. The main reason is that additional secondary antibodies could bind to primary antibodies from earlier staining rounds, increasing the signal of these primary antibodies, which would then be attributed to the wrong peak. For the validation experiments, we have used a simple correction method. In the 1st staining round, two identical samples were stained as usual. In the 2nd staining round, the control sample was stained with secondary antibodies only. The resulting signal increase in the control sample became the correction factor. Finally, for the calculation of the difference image, the pixel values of the 1st staining image were multiplied by this correction factor. In general, this method would require *n* − 1 control samples for *n* staining rounds.

**Fig. 5:**
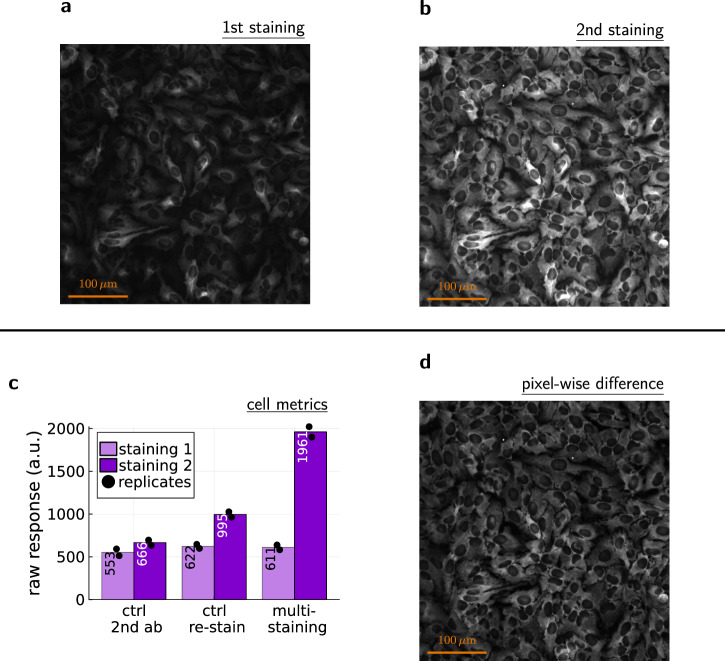
Illustration of the principle behind the binding-property-guided computational multiplexing. The same cell sample is stained twice with antibody concentrations determined from the accessibility histogram of a dilution series experiment. **a** Microscopy image after the 1st staining round with the lower antibody concentration. **b** Microscopy image after the 2nd staining round with the higher antibody concentration. **c** Signal intensities for the multi-staining and control wells (2 wells/replicates for each condition), quantifying 3000 cells (multiple distinct view fields) for each well. For “ctrl 2nd ab” only secondary antibodies were used for the 2nd staining round. For “ctrl re-stain” the 1st staining was repeated in the 2nd staining round, as additional control. **d** Pixel-wise difference (2nd staining image − 1st staining image). For the calculation of the pixel differences, the pixel values of the 1st staining image were multiplied by the increase-factor of “ctrl 2nd ab” to correct for the double-application of the same secondary antibodies. The difference image shows the fluorescence signals corresponding to the high-*K*_*τ*_ peak (azure, Fig. 4a) and the 1st staining image shows the fluorescence signal corresponding to the low-*K*_*τ*_ peak (orange, Fig. 4a).

Figure 5 shows the total signal images for the computational multiplexing validation experiment, together with the metrics for the total signal intensities (see Supplementary Fig. 3 for additional metrics). Aligning the images and subtracting a corrected 1st staining image from the 2nd staining image results in a difference image that contains only the signal corresponding to the high-*K*_*τ*_ peak (azure, Fig. 4a). The 1st staining image contains only the signal corresponding to the low-*K*_*τ*_ peak (orange, Fig. 4a).

**Fig. 6:**
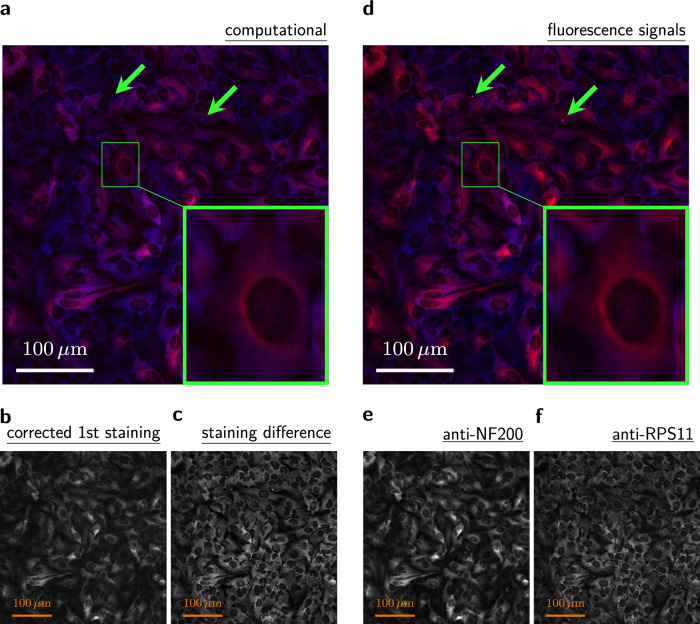
Result of the binding-property-guided computational multiplexing approach. **a** Composite image for the binding-property-guided computational multiplexing, using red for the 1st staining image (**b**) that corresponds to the low-*K*_*τ*_ peak (orange, Fig. 4a) and blue for the pixel-wise difference image (**c**) that corresponds to the high-*K*_*τ*_ peak (azure, Fig. 4a). **d** Composite image for the fluorescence-label signals after the 2nd staining, using red for the anti-NF200 label signal (**e**) and blue for the anti-RPS11 label signal (**f**). The green arrows point to debris that got assigned to the wrong peak.

For an easier comparison of the binding-property-guided computational multiplexing images with the individual antibody signals, we created color composite images. For the computational multiplexing composite image (Fig. 6a), red was used for the corrected 1st staining image (Fig. 6b) and blue was used for the difference image (Fig. 6c). For the fluorescence label composite image (Fig. 6d), red was used for the anti-NF200 signal image of the 2nd staining (Fig. 6e) and blue was used for the anti-RPS11 signal image of the 2nd staining (Fig. 6f). Here, we used the corrected 1st staining image for the computational multiplexing composite image, although the low-*K*_*τ*_ signal should generally be obtained from the uncorrected 1st staining image. This is necessary for a direct comparison between the composite images, because the fluorescence label composite image is created from the 2nd staining images, where secondary antibodies have already been applied twice. The correction for secondary antibodies essentially simulates this process for the 1st staining image.

The resulting composite images in Fig. 6 are almost identical. Furthermore, we have obtained additional view fields from two different wells and repeated the experiment, always obtaining similar composite images for the binding-property-guided computational multiplexing and the fluorescence labels (Supplementary Fig. 4). This demonstrates the validity of the binding-property-guided computational multiplexing, since the total signal removed all information about the individual antibodies.

It might be questioned whether accessibility histograms are needed for this multiplexing, as repeated staining of the same sample also works with arbitrary antibody concentrations. However, arbitrary antibody concentrations would lead to arbitrary signal splitting (Supplementary Figs. 5 and 6).

Despite the successful validation of the binding-property-guided computational multiplexing, there are limitations that need to be discussed. First, only fixed samples without movement between the staining rounds can be used. Second, any dust or debris that appears between the staining rounds can be attributed to the wrong peak (green arrows in Fig. 6a, d). Third, distinguishing between the signal contribution of distinct peaks in the accessibility histogram requires the existence of such peaks, which may not be the case for an antibody of interest.

## Discussion

In this paper, we have demonstrated applications of the accumulation model in the form of accessibility histograms. First, we have provided evidence that accessibility histograms describe the binding properties of antibodies. Second, we have shown that accessibility histograms can be used to determine optimal concentrations/dilution quotients for monoclonal antibodies that minimize the contribution of potentially unspecific background signals. Finally, we have illustrated how the binding information of accessibility histograms can be used to further investigate the epitope landscape with a simple multi-staining approach. The analysis methods for the accumulation model are implemented as ready-to-use Julia packages with documentation^22–27^.

The correspondence between accessibility histograms and binding properties may be used to investigate accessibility changes of epitopes that are caused by conformational changes or changes in the local epitope environment. This could offer a cheap and easy first analysis for detecting conformational changes with simple dose-response experiments. Since dose-response data points can always be achieved by simply repeating an experiment with different antibody concentrations, the accessibility analysis should be applicable to any immunoassay that allows a numerical quantification of the amount of bound antibodies. For example, repeating a Western Blot experiment with different antibody concentrations could lead to a correlation of signal intensity with protein weight/size and epitope binding rate/accessibility.

Although the selection of optimal staining concentrations from accessibility histograms is restricted to monoclonal antibodies, the underlying principle enables a binding-property-guided computational multiplexing. The idea of repeated staining rounds might appear similar to the label coding described in refs. ^28–30^, where a label response code is obtained from sequential imaging, but the working principle is fundamentally different. In our approach, multiplexing arises from the binding-property-heterogeneity of epitopes. Because of this, our computational multiplexing could be well-suited for the investigation and application of bi-specific antibodies. So far, bi-specific antibodies seem to be of interest only for therapeutics^31–33^, as simultaneous staining of multiple targets is not desirable for immunostaining antibodies. Hence, modeling approaches for bi-specific binders often have a therapeutic focus or investigate general binding properties^31–36^. Accessibility histograms could help in the research of bi-specific antibodies. Going one step further, the binding-property-guided computational multiplexing could even render bi-specific antibodies viable for immunostaining, allowing for a single-label multiplexing of different targets.

Despite providing replicable and consistent results for the applications that we have shown, the underlying model is only an empirical model. For example, neither the effects of antibody depletion nor the dynamics of secondary antibodies are modeled. However, a major issue for extended models is that additional parameters to account for these effects are hardly measurable.

Although depletion can be modeled, the superposition of epitope classes leads to a coupled dynamical system without analytical solution, requiring parameter estimation with numerical solutions, which is beyond the scope of this paper. Furthermore, unit conversion factors do not cancel out when antibody depletion is considered (Supplementary Note 3.3). However, although we do not model antibody depletion in this paper, we suggest a simple worst-case correction for depletion effects similar to the concentration correction described in ref. ^37^ (see Supplementary Note 4).

Furthermore, it should be noted that any information provided by the accessibility histogram depends on the dose-response curve. On the one hand, this means that the accessibility histogram can only be taken seriously if one can assume that the dose-response curve is characteristic of the system under investigation. Hence, using only one replicate that may be affected by strong noise/biological variability, or data with severe outliers, can produce wrong results. On the other hand, the accessibility histogram is incomplete/inconclusive when the dose-response curve does not capture the entire binding behavior of the antibody. If the dose-response curve does not start from the baseline, low-*K*_*τ*_ peaks may be missed. However, this can easily be fixed with additional dilution steps. The opposite case, a dose-response curve that is flat and close to the baseline except for a few data points, occurs when the highest concentration/dilution quotient is not high enough. Often, this cannot be fixed easily, either because the antibody stock solution is not concentrated enough or because higher antibody concentrations are too expensive. In this case, the histogram will show only a single peak, clipping to the right of the histogram, which does not provide any meaningful information. However, this scenario may be rephrased as a warning sign that the tested concentrations do not provide enough information to distinguish epitope classes.

## Methods

### Cell culture

HeLa cells were obtained by the DSMZ-German Collection of Microorganisms and Cell Cultures GmbH and cultured in growth medium (DMEM with 1% penicillin-streptomycin 10^4^ U/mL solution and 10% fetal calf serum) at 37 °C and 5% CO_2_ in the incubator. For passaging and seeding, the cells were washed with PBS and incubated with trypsin for 2 min before adding fresh medium and subsequent centrifugation. After removing the supernatant, the cells were re-suspended with fresh medium. For the seeding, the cells were counted with a Neubauer-ruled hemocytometer and diluted with medium to obtain a cell density of approximately 10^8^ L^−1^. Then 100 μL of the cell solution was added to each well of a *μ*Clear 96-well plate, leading to approximately 10^4^ cells per well.

### Mouse DRG neurons

Male C57Bl/6N mice aged 1–3 months were obtained from Charles River Laboratories. Mice were kept in a temperature- and humidity-controlled animal care facility in IVC-system cages as recommended by the German Society of Laboratory Animal Science at the University Hospital of Cologne on a 11-h light/dark cycle and provided with food and water *ad libitum*. Mice were scarified by inhalation of CO_2_ followed by decapitation. DRGs (cervical, lumbar, and thoracic) were removed within 30 min per animal. No experiments were performed on living animals. We have complied with all relevant ethical regulations for animal use. All experiments were performed in accordance with the German animal welfare law with permission of the District Government for Nature and Environment, NRW (approval registered as UniKöln_Anzeige Section 4.18.003 and UniKöln_Anzeige Section 4.23.005).

### Stimulation and fixation

After seeding the HeLa cells, the cell plates were incubated for 24 h before cAMP-stimulation (only applicable for anti-pRII antibody experiments) and fixation.

For the cAMP-stimulation 10 μM Sp-8-Br-cAMPS-AM was used per well. For this, Sp-8-Br-cAMPS-AM was diluted in DMSO and mixed with medium, taken from the cell plate. The mixing was done in a separate 96-well V-bottom plate on a 37 °C-pre-warmed thermal pad. Then the mixture was re-applied from the V-bottom plate back to the cell plate. Afterwards, the cells were incubated at 37 °C and 5% CO_2_ for 15 min. Immediately after the incubation, the cells were fixed.

For the fixation, 100 μL of an 8% Paraformaldehyde (PFA) solution were added to the 100 μL of medium (also containing stimulation compounds if applicable) that were already present in the well, leading to a 4% PFA solution. After 5  min, half of the well-content was removed, and after another 5 min the cells were washed with PBS.

When the plates were not used immediately, they were sealed and stored in a fidge at 4 °C.

### Immunocytochemistry

The cells were permeabilized and blocked with 2% normal goat serum, 1% BSA, 0.1% Triton X-100, and 0.05% Tween 20, all dissolved in PBS, for 1 h at room temperature. The primary antibodies were diluted in PBS with 1% BSA in a separate 96-well V-bottom plate. After removing the blocking/permeabilization solution and applying the primary-antibody solution, the cell plates were stored in the fridge at 4 °C for 24 h.

The next day, the cells were washed three times with PBS. The plates were kept at room temperature for 10 min between the washings. Then, secondary antibodies and Hoechst, diluted 1–1000 in PBS, were applied. After this, the plates were kept in a dark environment for 1 h at room temperature. Finally, the cells were washed 3 times with PBS, again with 10 min between the washings.

#### Incubation time experiments

For the incubation time experiments, the following stains, primary antibodies, and secondary antibodies were used:

- **anti-NF200**: mouse monoclonal, anti-Neurofilament 200, Sigma-Aldrich, N0142-.2ML
- **Hoechst** 34580: Sigma-Aldrich, 63493
- **Alexa Fluor Plus 647**: goat polyclonal anti-mouse, Invitrogen, #A32728

#### Antibody mixture experiments

For the antibody mixture dose-response experiments and the multi-staining experiments, the following stains, primary antibodies, and secondary antibodies were used:

- **anti-NF200**: mouse monoclonal, anti-Neurofilament 200, Sigma-Aldrich, N0142-.2ML
- **anti-RPS11**: rabbit monoclonal, anti-Ribosomal protein S11/RPS11, Abcam, ab175213
- **Hoechst** 34580: Sigma–Aldrich, 63493
- **Alexa Fluor Plus 647**: goat polyclonal anti-mouse, Invitrogen, #A32728
- **Alexa Fluor 555**: goat polyclonal anti-rabbit, Invitrogen, #A-21429
- **Alexa Fluor 555**: goat polyclonal anti-mouse, Invitrogen, #A-21424 (used for normalization controls)

To achieve higher concentrations of anti-NF200 antibody compared to anti-RPS11 antibody, the anti-NF200 antibody was diluted 1 to 20 from the vendor stock, while the anti-RPS11 antibody was diluted 1 to 100 from the vendor stock. This applies to the antibody mix and to the individual antibodies if used separately. The resulting “initial dilution solution” was then always given the dilution quotient 1:100 (even when only the anti-NF200 antibody was used, to ensure that it was indeed more concentrated). Note that the choice 1:100 for the dilution quotient of the initial dilution solutions is completely arbitrary. We chose it merely because we started with anti-RPS11 at a 1-to-100 dilution (approximately a dilution quotient of 1:100), which we henceforth used as a reference point.

#### PKA experiments

For the PKA experiments the following stains, primary antibodies, and secondary antibodies were used:

- **anti-pRII**: rabbit monoclonal, anti-PKA R2/PKR2 (phospho S99), Abcam, ab32390
- **Hoechst** 34580: Sigma-Aldrich, 63493
- **Alexa Fluor Plus 647**: goat polyclonal anti-rabbit, Invitrogen, #A32733
- **anti-(UCH-L1/PGP9.5)** antibody (Novus, NB110-58872) **(only for mouse DRG neurons)**

### Data quantification

All plates were scanned with a laser-illuminated High Content Screening (HCS) microscope (Cellinsight CX7 LZR, Thermo Scientific). The accompanying HCS Studio (v6.6.2) software was used to simultaneously capture the images and quantify the fluorescence signals. For quantification, cells were identified with Hoechst staining, and cell masks were defined by increasing the identified nucleus regions (example shown in Supplementary Fig. 7c). The HCS Studio software calculated the measured average fluorescence intensity for the individual cells (within the cell mask region) for each channel.

For the antibody mixture dose-response experiments and multi-staining experiments, images were captured until 3000 distinct cells were quantified per well, with a hard limit of 50 images per well (preventing hour-long scanning of an accidentally empty well). Most wells reached 3000 distinct cells, with all but two wells above 2000 quantified cells (two exceptions with 1851 and 1993 cells, respectively). For the incubation time experiments, the cell target was 4000 (reached except for two wells: 2619 and 2786 cells, respectively). For the PKA experiments, the cell target was 5000 (always reached).

For the antibody-mixture dose-response experiments, the exposure times were almost identical (for one plate, 0.022 s instead of 0.025 s for the Alexa Fluor Plus 647 channel, to avoid signal clipping). For the incubation time experiments and the PKA experiments, the exposure times were identical for the respective plates. The multi-staining experiments used the same exposure times for both staining rounds. However, the two plates were independent experiments using different exposure times.

The raw monochrome images (unedited images) for each channel were exported as PNG files for the antibody mixture dose-response experiment and as 16-Bit Tiff files for the multi-staining experiments. The single-cell data was exported as CSV file for each plate. Subsequent data analysis and image editing were performed with Julia (v1.9.1).

### Data normalization and dose-response curves

Different fluorescence labels were used for the antibody-mixture dose-response experiments (Alexa Fluor 555 and Alexa Fluor Plus 647). Furthermore, one plate used a slightly different exposure time for Alexa Fluor Plus 647. Thus, a normalization condition (multiple wells) was used on each plate to normalize the signal intensities between the different plates and labels. This normalization condition consisted of mouse anti-NF200 (diluted 1 to 1000 from the vendor stock solution) as primary antibody and anti-mouse Alexa Fluor 555 as well as anti-mouse Alexa Fluor Plus 647 as secondary antibodies. Since the exposure times were identical for all PKA plates and all incubation time plates, and since only a single channel was used for these experiments, the zero control (using only secondary antibodies) was used for the normalization between different plates.

To obtain the normalization value, the well intensity (mean value of individual cell signal intensities) was calculated for each normalization well and each fluorescence label individually. The mean value of the well intensities was used as normalization value for the respective plate and the respective channel. To normalize the signal intensities of a plate, the signal intensity of each cell was divided by the respective normalization value. (Instead of normalizing the single-cell intensities, it would be equally possible to just normalize the dose-response values at the end. Since the normalization is a linear transformation, the result would be identical up to floating point errors.)

After normalizing the single-cell signal intensities, the average signal intensity was calculated for each well and each fluorescence signal. For the dose-response curves, the wells were considered as replicates. The mean of the well intensities was used as data point, and the standard deviation was used as measurement uncertainty. Finally, for each dose-response curve, the non-zero baseline signal was removed. For this, we used the baseline obtained from the zero controls. However, to prevent negative values, we used the lowest response value of the dose-response curve if subtracting the baseline would result in negative response values.

### Model fitting

We developed and used Julia packages^22–27^ for all accessibility analyses. Here, we mostly give a high-level description, not subject to the actual implementation of the packages. For instructions how to implement the methods described in this section and for a complete list of the available options, please refer to the extensive documentation of the packages.

#### Fitting objective

Let ${\{({d}_{i},{r}_{i},\Delta {r}_{i})\}}_{i=1}^{n}$ denote the dilution quotients, the response values (mean of replicates) and the measurement uncertainties (standard deviation of replicates) of a dose-response curve, then the objective function that was used for the curve fitting reads:

$$\,obj\,(\lambda )={\ell }_{0}(\lambda )+{\sum }_{i=1}^{n}\frac{{({r}_{i}-f({d}_{i},\lambda ))}^{2}}{{(2\Delta {r}_{i})}^{2}}\,.$$

This is a sign-flipped logarithmic posterior distribution that assumes the measured responses to be independent and normally distributed. Here, *λ* denotes the parameters for the model function *f*, and *ℓ*_0_ denotes the sign-flipped logarithmic prior.

Flipping the sign is only an implementation detail of the Julia packages, such that both least-squares and (logarithmic) posterior fitting lead to objective functions that need to be minimized.

Since the measurement uncertainties appear in the denominator, *Δr*_*i*_ = 0 is ill-defined. But also, *Δr*_*i*_ > 0 that are sufficiently small can cause problems, as they dominate the resulting objective function, leading to poor fitting results after numerical optimization. In either case, the standard deviation must be replaced by a sufficiently large value to obtain a well-behaved fitting objective function. This became necessary for the cAMP-stimulated HeLa cells, where we replaced the standard deviation of the lowest concentration (zero due to baseline removal) with 0.01.

Both the model function and the logarithmic prior depend on the grid discretization of the *K*_*τ*_-domain that is used for the numerical approximation of the integral in Eq. (2). Thus, let {*p*_1_, …, *p*_*m*_} denote the discretization points of the *K*_*τ*_-domain, which define the intervals:

$$[{p}_{1},{p}_{2}),[{p}_{2},{p}_{3}),\ldots ,[{p}_{m-1},{p}_{m})\,.$$

The parameters in *λ* = (*λ*_1_,…,*λ*_*m*−1_) are the amounts of equivalent epitopes with *K*_*τ*_ in the respective interval.

For a given grid, we used the following model function to approximate the integral model (2):

$$f(d,\lambda )={\sum }_{j=1}^{m-1}{\lambda }_{j}\left(1-\exp \left(-\frac{d}{({p}_{j+1}+{p}_{j})/2}\right)\right)\,.$$

Note that *g*(*p*) in (2) is in fact the epitope density, not the amount of epitopes. Thus, its discretization should be $\frac{{\lambda }_{j}}{{p}_{j+1}-{p}_{j}}$, but the factor $\frac{1}{{p}_{j+1}-{p}_{j}}$ cancels out with the factor *p*_*j*+1_ − *p*_*j*_ from the Riemann sum approximation.

For the sign-flipped logarithmic prior, we chose the prior probability so that it essentially becomes a Tikhonov regularization for the differences *λ*_*i*+1_ − *λ*_*i*_ of the amounts of epitopes with *K*_*τ*_ in neighboring intervals. However, the differences were rescaled with respect to the visual log-scale area of the histogram plots:

$${\ell }_{0}(\lambda )=\frac{\alpha }{{m}^{2}}{\sum }_{j=1}^{m-2}{\left(\frac{{\lambda }_{j+1}}{\log ({p}_{j+2})-\log ({p}_{j+1})}-\frac{{\lambda }_{j}}{\log ({p}_{j+1})-\log ({p}_{j})}\right)}^{2},$$

where $\log$ is the logarithm with base 10 and *α* is the smoothness regularization parameter. For all analyses we used *α* = 500 as default parameter and *α* = 50 for the weak regularization plots (Supplementary Figs. 1 and 2).

#### Adaptive grids and objective function minimization

We used adaptively refining grids for the minimization of the objective function. First, a coarse logarithmically spaced two-interval grid (three discretization points) was defined:

$$\begin{array}{rcl}{p}_{1} & = & \min \{{d}_{i}\},\,\log ({p}_{2})=\frac{\log (\max \{{d}_{i}\})+\log (\min \{{d}_{i}\})}{2},\\ {p}_{3} & = & \max \{{d}_{i}\}\,.\hfill\end{array}$$

Then, the corresponding objective function was constructed, as explained above, and minimized with the gradient-free, box-constrained Nelder–Mead algorithm (of the Optim.jl package) using 2000 iterations. After obtaining the parameters *λ* from the numeric minimization, the contribution value of each interval was defined (cf. Supplementary Note 2.2) by

$$\frac{{\lambda }_{j}}{\log ({p}_{j+1})-\log ({p}_{j})}\,.$$

To refine the grid adaptively, the interval with the largest contribution value difference, compared to its neighbors, was split into two. Each of the intervals got $\frac{{\lambda }_{j}}{2}$ as new parameter (equal distribution of the amount of equivalent epitopes). After splitting the interval, the objective function was constructed for the now refined grid. Using the correspondingly refined parameters as new starting point, the objective was minimized again.

This optimization-refinement cycle was repeated 50 times to obtain a sufficiently fine grid. After the last refinement, the refined parameters were optimized with the box-constrained LBFGS algorithm (of the Optim.jl package), using numerically calculated gradients with an absolute tolerance of 1e-12 and 2000 iterations.

### Plotting histograms

To plot the parameters {*λ*_1_, …, *λ*_*m*−1_} that correspond to a grid {*p*_1_, …, *p*_*m*_} as a histogram, bars are defined according to the intervals [*p*_1_, *p*_2_), [*p*_2_, *p*_3_)…. Then, for each bar, its width *w*_*i*_ on a logarithmic scale is calculated by

$${w}_{i}=\log ({p}_{i+1})-\log ({p}_{i})\,.$$

The height of a bar in the histogram is then the parameter divided by this logarithmic width *λ*_*i*_/*w*_*i*_. Finally, the bars are plotted on a logarithmic scale.

### Image processing for Fig. 4b, c

The raw monochrome PNG images for the individual channels were imported into Julia, essentially represented as matrices of Float64 values. To normalize the image brightness, the quotient of the normalization values for the respective plate was calculated (cf. “Data normalization”):

$$r=\frac{norm.\,{value}_{Alexa}\,Plus\,647}{norm.\,{value}_{Alexa}\,555}.$$

This quotient describes the factor by which the Alexa Fluor Plus 647 signal is stronger than the Alexa Fluor 555 signal, all else being equal (by assumption of the normalization control setup). Thus, each pixel intensity of the Alexa Fluor 555 image matrices was multiplied with *r*. All pixel intensities were multiplied by a factor of 30 to increase the overall brightness for better visibility. The composite images were created by adding the monochrome images for the fluorescence labels pixel-wise, assigning the following colors (i.e., multiplying the pixel intensity with the respective color vector):

**Table:** 

Finally, the matrices for the fluorescence label images and the composite image were exported as PNG files with a 2 × 2 pixel-binning and clipping of too high intensities.

### Image processing for Supplementary Fig. 7a, b

The images were processed in the same way as for Fig. 4b, c. However, the images were only brightened by a factor of 20 (instead of 30). In addition, the Hoechst staining was included. The Hoechst signal was not normalized, but all pixel intensities were multiplied by a factor of 4, so that the Hoechst staining is visible but not distracting in the composite images. Then, the composite images were created, and as before, with the following colors:

**Table:** 

### Multi-staining

The 1st staining round of the multi-staining experiments followed the protocol described in the “Immunocytochemistry” section. Both the actual multi-staining condition and the controls were stained with the primary-antibody mix (1-to-20 dilution of anti-NF200 and 1-to-100 dilution of anti-RPS11). In all cases, a 1-to-1000 dilution of secondary antibodies/stains (anti-mouse Alexa Fluor Plus 647, anti-rabbit Alexa Fluor 555, and Hoechst) was used. After the immunocytochemistry, the prepared wells were scanned with the HCS microscope.

Immediately after scanning the plate, the 2nd staining round was commenced, using the same protocol as before, but not repeating the blocking. For the multi-staining condition, we used the dilution quotient *d*_2*nd*_ = 1:6400 for the primary-antibody mix. For the re-stain control “ctrl re-stain”, we used the same dilution quotient as used for the 1st staining, and for the secondary-antibody control “ctrl 2nd ab”, the wells were left untouched. After the 24 h primary-antibody incubation, the secondary antibodies but not the Hoechst stain were applied at the usual 1-to-1000 dilution to all conditions. After this, the wells were scanned again, using exactly the same settings as for the first scanning.

The raw images were exported as 16-bit TIFF images to retain all data. To align the images of the two different scans, we compared the respective Hoechst signals. With the SubpixelRegistration.jl package, we determined the pixel shifts that are necessary to align the Hoechst channel images. Then, we applied the same pixel shifts to the respective images for the other fluorescence labels and cropped all images to the overlapping region. For the rest of the image processing, the Hoechst images were not used. Furthermore, the brightness (pixel intensities) of all images was increased by a factor of 6 just before exporting the images (after the edits described below), to increase the visibility. Note that no channel normalization was applied here, since all wells were prepared on the same plate (for one independent repetition of the experiment), and since the relative brightness of the fluorescence labels does not affect the result (same effect on computational multiplexing and the fluorescence label validation).

We exported the fluorescence-label images of the 2nd staining without any additional editing as monochrome images (Fig. 6e, f). The corresponding composite images (Fig. 6b and Supplementary Fig. 4) were calculated as explained in the “Image processing for Supplementary Fig. 7a, b” section, without the Hoechst channel.

For the computational multiplexing, we removed the fluorescence label information by adding together the pixel intensities of the monochrome images. This resulted in a single, total-signal-intensity, monochrome image per staining and removed the fluorescence-label information that would not be present in generic multi-staining experiments. From the HCS microscope data, we determined the fold-change of the secondary-antibody control and increased the brightness of the 1st staining image by this fold-change. Next, we calculated the pixel-wise difference image between the 2nd staining image and the brightness-increased 1st staining image. All images were exported as monochrome images (Fig. 5a, b, d and Fig. 6b, c). Finally, we created a composite image (Fig. 6a) as done before, using the colors RGB (1, 0, 0) for the brightness-increased 1st staining image and RGB (0,0, 1) for the difference image.

### Reporting summary

Further information on research design is available in the Nature Portfolio Reporting Summary linked to this article.

## Statistics and reproducibility

Except for the calculation of data points (mean values) and sample standard deviations (error bars), no statistical analysis was applied.

### Incubation time experiments

For each incubation time, 16 individual dilution series (on two separate 96-well plates) were prepared, leading to 16 replicates.

### Antibody mixture experiments

For the dose-response experiments, four 96-well plates were used, containing the dilution series for the anti-NF200 antibody (1x), the anti-RPS11 antibody (1x), and the antibody mixture (2x), leading to four replicates for the individual antibodies and eight replicates for the antibody mixture. However, two replicates were removed completely because of significant outliers and to avoid cherry-picking of data points.

### PKA experiments

Unstimulated and cAMP-stimulated cells were prepared on the same 96-well plate, leading to a dilution series for each condition. In total, three plates and thus three replicates were prepared.

### Multi-staining

For each condition, control, etc., two wells were prepared, and multiple images from different regions in the well were captured. The whole experiment was once repeated independently.

## Supplementary information

Supplemental Information

Reporting Summary

## Data Availability

All data (single-cell data and TIFF-images for the multi-staining) are available at^38^.

## Code availability

The analysis methods for the accumulation model are implemented as ready-to-use Julia packages with documentation^22–27^. Auxiliary code to process the data and images for the multi-staining can be found in the data repository^38^.
