# Supplementary material for: Computational epitope heterogeneity analysis in immunostainings from antibody-dilution series
Source: Commun Biol. 2026 Feb 2;9:238. doi: 10.1038/s42003-026-09517-x (PMC12902027; doi:10.1038/s42003-026-09517-x)
Supplement: Supplementary file 1 — Supplemental Information [file 42003_2026_9517_MOESM1_ESM.pdf]

# Contents


**1 Extended Data Figures** 2

**2 The accessibility histogram and its visualization** 9

2.1 Histograms as approximation of the accessibility density . . . . . . . . 9

2.2 Visualization and dose-response contribution . . . . . . . . . . . . . . 10

**3 The accessibility histogram does not depend on the choice of units** 12

3.1 Measurement limitations and unknown proportionality factors . . . . . 13

3.2 Unit conversion factors cancel out in the discrete accumulation model 13

3.3 Conversion factors do not cancel out for the antibody depletion model 14

3.4 Choice of units and the accessibility histogram . . . . . . . . . . . . . 16

**4 Depletion correction** 19

**5 Anti-pRII in mouse DRG neurons** 22

**6 Choice of regularization parameter** 24

**7 Data uncertainties and fitting tips** 26

**8 Additional regularization-parameter plots** 28

8.1 Incubation time experiments: 10 min incubation . . . . . . . . . . . . . 28

8.2 Incubation time experiments: 1 h incubation . . . . . . . . . . . . . . . 30

8.3 Incubation time experiments: 21:20 h incubation . . . . . . . . . . . . 32

8.4 Histogram validation: Anti-Nf200 . . . . . . . . . . . . . . . . . . . . . 34

8.5 Histogram validation: Anti-RPS11 . . . . . . . . . . . . . . . . . . . . 36

8.6 Histogram validation: Antibody mix . . . . . . . . . . . . . . . . . . . 38

8.7 Anti-pRII antibody: Unstimulated HeLa cells . . . . . . . . . . . . . . 40

8.8 Anti-pRII antibody: cAMP-stimulated HeLa cells . . . . . . . . . . . . 42

8.9 Anti-pRII antibody: Unstimulated DRG neurons with uniform errors . 43

8.10 Anti-pRII antibody: cAMP-stimulated DRG neurons with uniform errors 45

8.11 Anti-pRII antibody: Unstimulated DRG neurons . . . . . . . . . . . . 48

8.12 Anti-pRII antibody: cAMP-stimulated DRG neurons . . . . . . . . . . 50

**9 Removed replicates** 51

# 1 Extended Data Figures

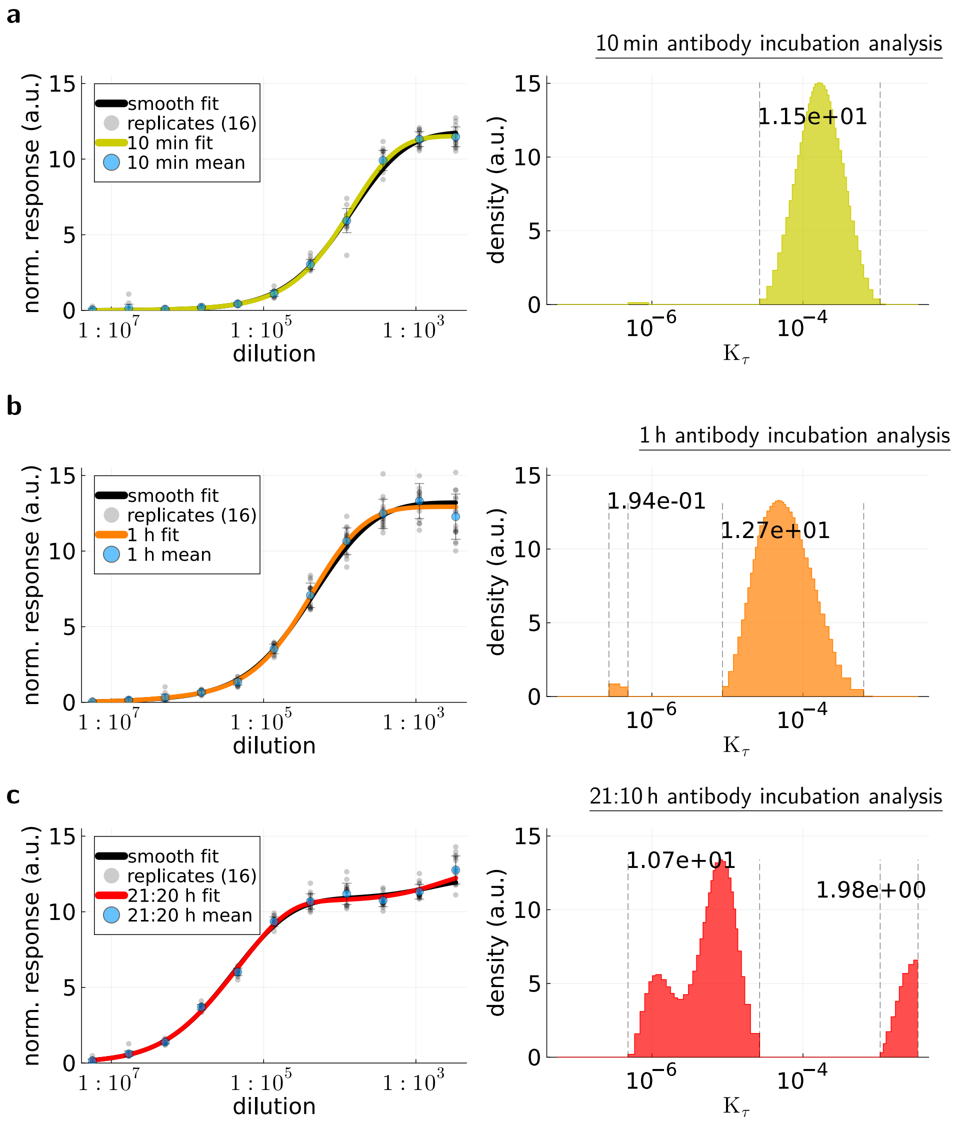


**Supplementary Figure 1**: Dose-response curves (same data as main Fig. 1d-f; mean values of $n = 16$ replicates; error bars = sample std) and the corresponding accessibility histograms obtained with weaker regularization ($\alpha = 50$ instead of $\alpha = 500$, cf. Methods). The smooth fit (black line) in the dose-response plots shows the model curve that was obtained for $\alpha = 500$.

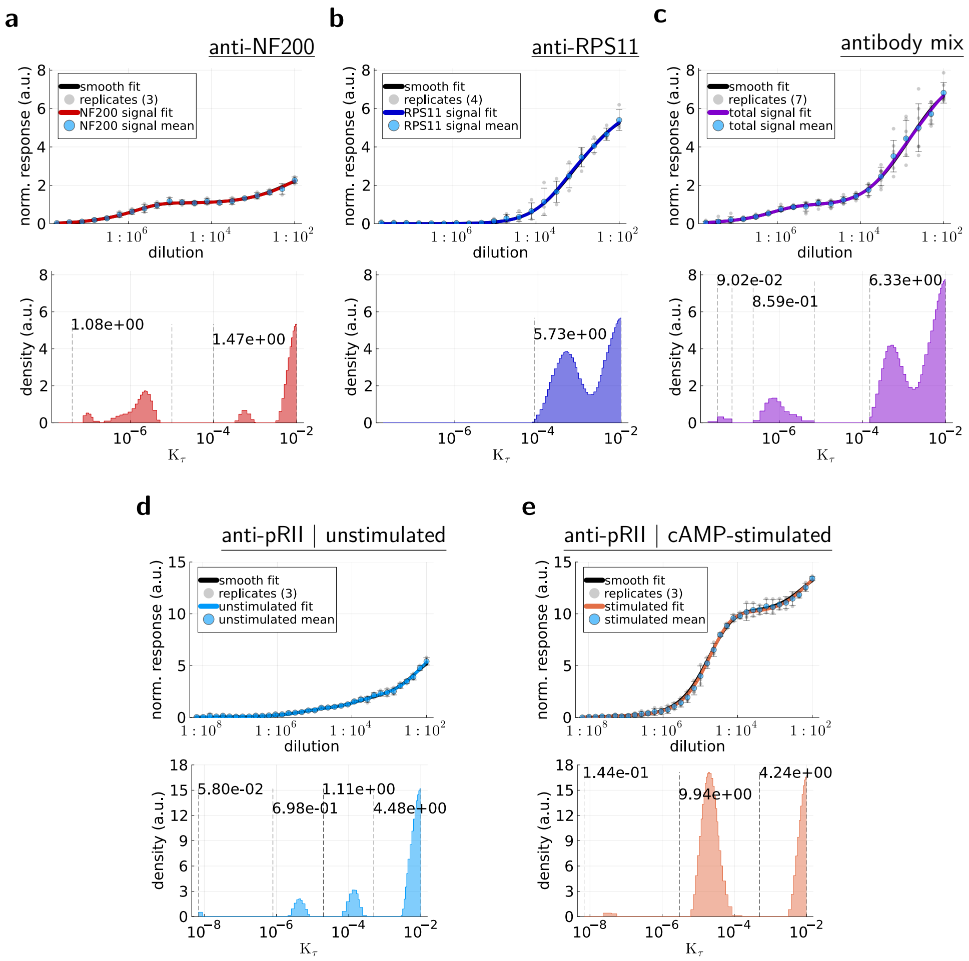


**Supplementary Figure 2**: Dose-response curves (same data as main Fig. 2; mean values of replicates; error bars = sample std) and the corresponding accessibility histograms obtained with weaker regularization ($\alpha = 50$ instead of $\alpha = 500$, cf. Methods). The smooth fit (black line) in the dose-response plots shows the model curve that was obtained for $\alpha = 500$.

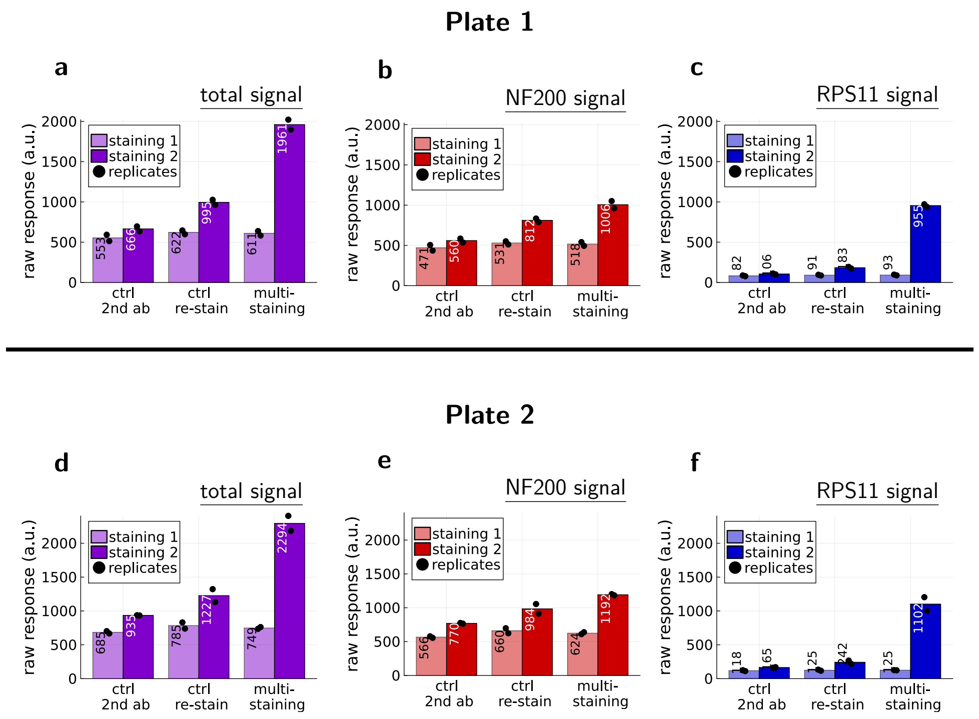


**Supplementary Figure 3**: Signal intensities for the multi-staining and control wells (2 wells/replicates for each condition), quantifying 3000 cells for each well. For "ctrl 2nd ab", only secondary antibodies were used for the 2nd staining, and for "ctrl re-stain", the 1st staining was repeated in the 2nd staining round.

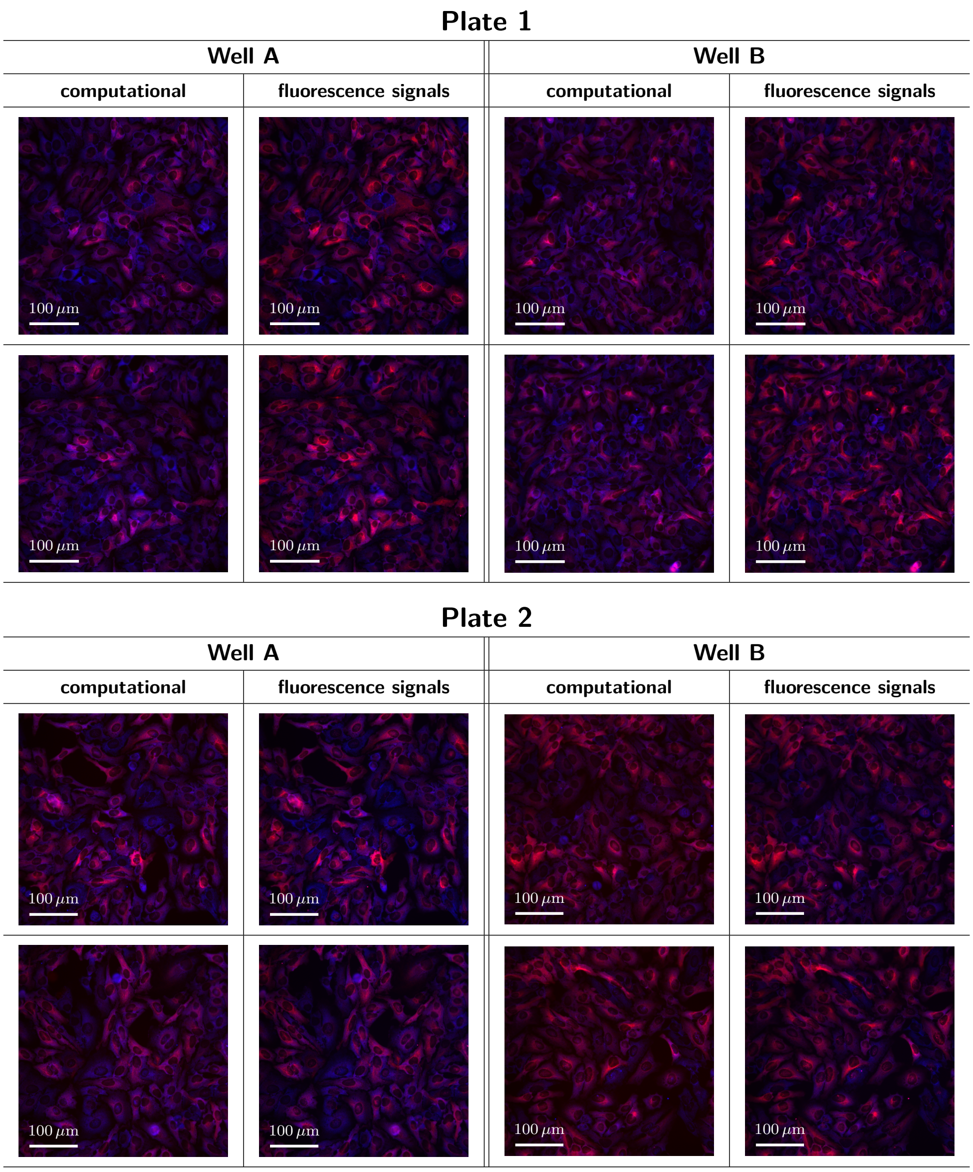


**Supplementary Figure 4**: Additional computational multiplexing composite images and fluorescence-signal composite images from both plates. On each plate, 2 separate wells were used for the multi-staining. For each well, multiple distinct view fields were captured.

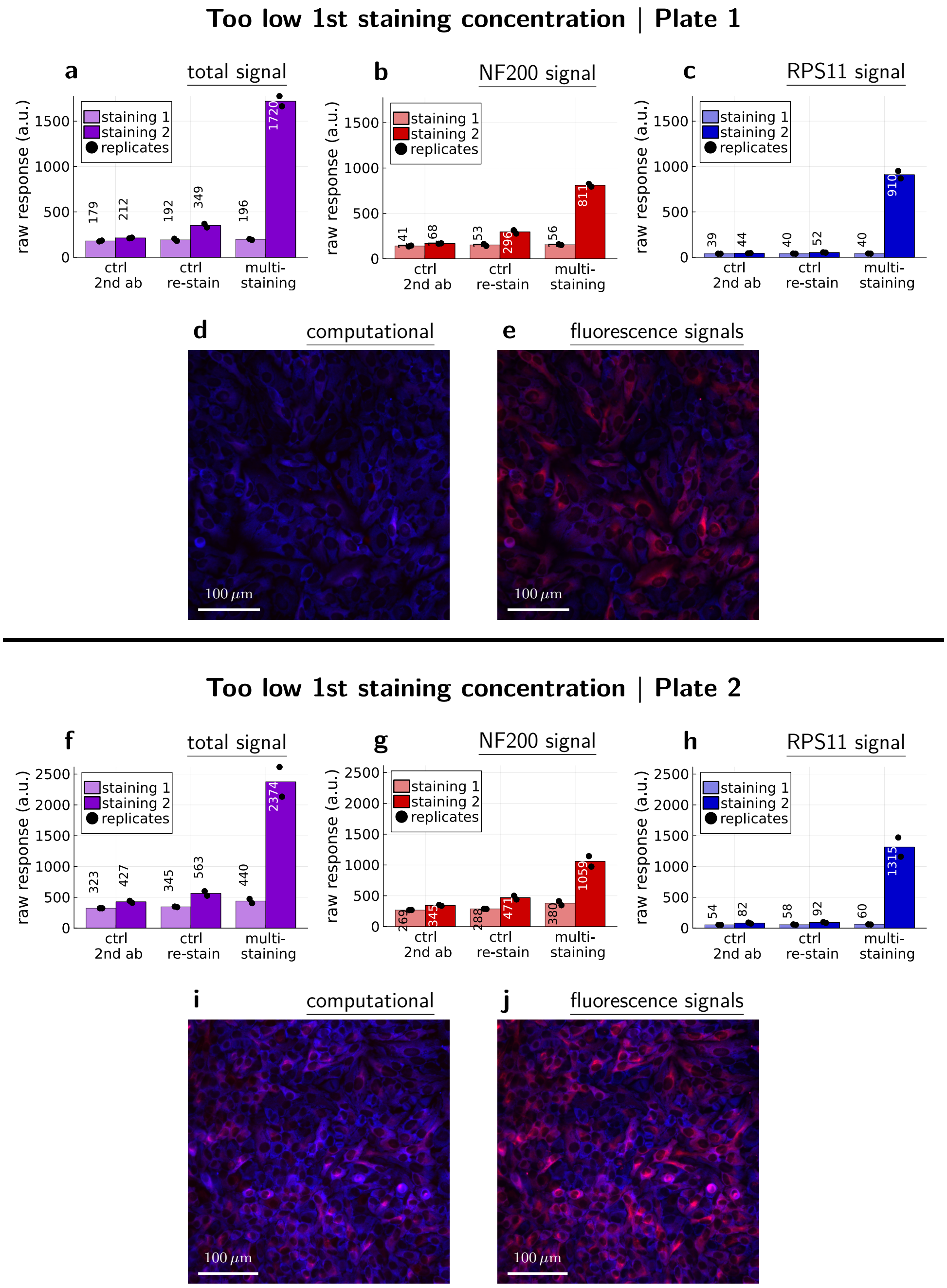


**Supplementary Figure 5**: Computational multiplexing experiment, using a too low dilution quotient (1 : 1638400 instead of 1 : 102400) for the 1st staining. The computational multiplexing composite images attribute too much of the signal to the high-$K_\tau$ peak.

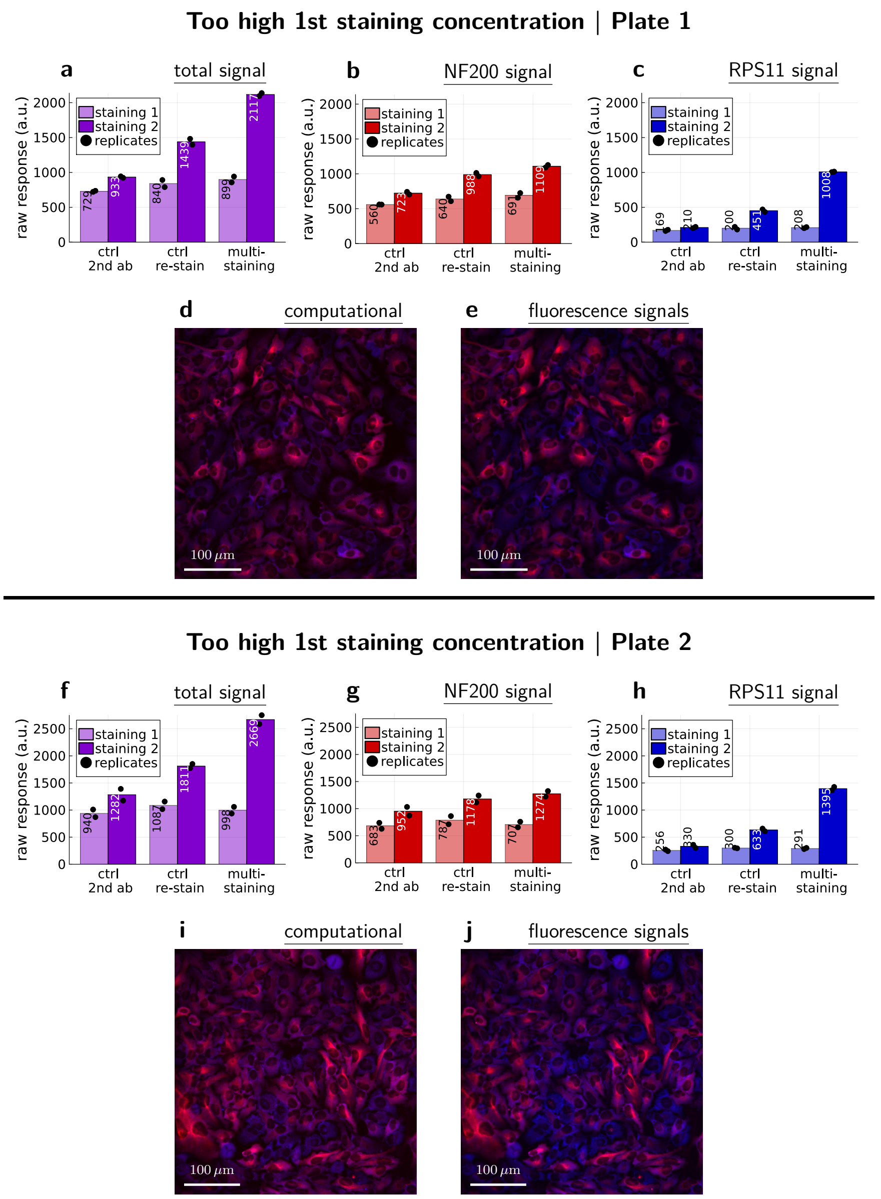


**Supplementary Figure 6**: Computational multiplexing experiment, using a too high dilution quotient (1 : 25600 instead of 1 : 102400) for the 1st staining. The computational multiplexing composite images attribute slightly too much of the signal to the low-$K_\tau$ peak. This effect is weak, as the low dilution quotient for the 2nd staining round (1 : 6400) prevented the use of much higher 1st staining dilution quotients.

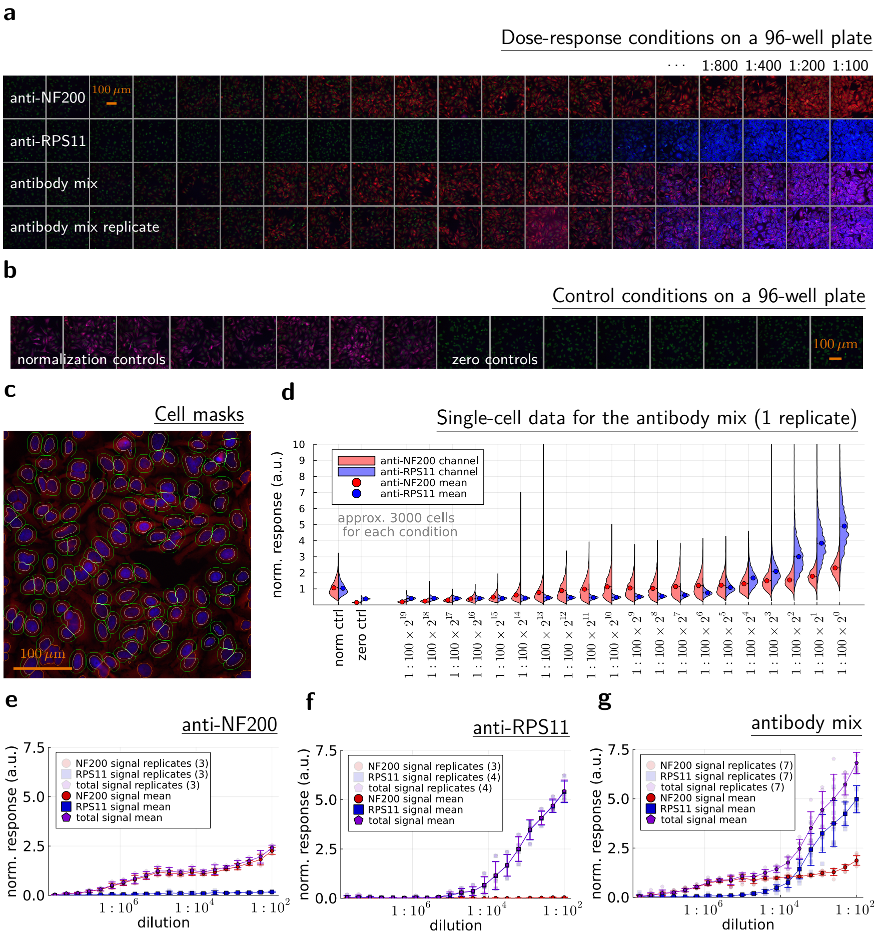


**Supplementary Figure 7**: **a**,**b**, Example images of the dilution series (a) and control wells (b) on a single plate for the antibody-mix dose-response experiments. The total brightness of the images was uniformly increased for better visibility. The colors do not represent the fluorescence emission frequencies. They were chosen for better visibility in case of red-green blindness. **c**, Example image of the automatic quantification from the High Content Screening microscope software. Cell nuclei were identified with the Hoechst staining (here blue instead of green for better visibility in case of red-green blindness). The quantification regions (green circles) were defined by increasing the nuclei regions (white circles). Nuclei regions too close to the edges, regions that are deformed etc., were excluded (orange circles). **d**, Distribution of the cell signal-intensities for a single antibody-mix replicate. All signal intensities were normalized to the average response of the normalization controls. **e**-**g**, Dose-response curves (mean values of replicates ($n$ indicated in legends), error bars = sample std) for the anti-NF200 antibody (e), the anti-RPS11 antibody (f) and the antibody mix (g). In each case, all fluorescence signals were measured (red = anti-NF200, blue = anti-RPS11, purple = total signal).

# 2 The accessibility histogram and its visualization

Approximating the sum of the discrete accumulation model (1) by an integral removes the need to determine the unknown number of epitope classes. Yet, a generic continuous function is difficult to represent in a computer and is thus difficult to use for inverse problems. Hence, an approximation of the density with a finite set of parameters is required.

## 2.1 Histograms as approximation of the accessibility density

Recall that the accessibility density function $g(K_\tau)$ is in fact just an approximation of a finite set of epitope classes $\{(g_i, K_{\tau,i})\}$. Since physically meaningful binding rates should be finite, there are finite values $p_{\min}$ and $p_{\max}$ such that

$$0 < p_{\min} \leq K_{\tau,i} \leq p_{\max} < \infty \qquad \forall\, i \in \{1, \dots, N\}\ .$$

In other words, for any density function $g(K_\tau)$ that describes a real system, there is a smallest positive accessibility constant $p_{\min} > 0$ and a largest finite accessibility constant $p_{\max} < \infty$ for which $g(K_\tau) \neq 0$.

As described in the Methods section, the accessibility density function $g(K_\tau)$ is approximated by constant functions on a finite grid. For this grid, $m-2$ additional discretization points $\{p_1, \dots, p_m\}$ between $p_1 = p_{\min}$ and $p_m = p_{\max}$ are defined, such that

$$p_i \leq p_j \qquad \forall\, i < j\ .$$

These discretization points define the following intervals:

$$I_1 = [p_1, p_2), \quad I_2 = [p_2, p_3), \dots\ , \quad I_{m-1} = [p_{m-1}, p_m)\ .$$

An approximation of $g(K_\tau)$ with constant functions on the intervals $\{I_j\}_{j=1}^{m-1}$ then reads

$$g(K_\tau) \approx \sum_{j=1}^{m-1} g\left(\tfrac{p_{j+1}-p_j}{2}\right) \chi_{I_j}(K_\tau)\ , \qquad \text{where} \qquad \chi_{I_j}(K_\tau) = \begin{cases} 1 & ,\ K_\tau \in I_j \\ 0 & ,\ K_\tau \notin I_j \end{cases}\ .$$

In case of an unknown accessibility density function that is to be inferred form data, the approximation consists of $m-1$ unknown parameters $\lambda_1, \dots, \lambda_{m-1}$:

$$g(K_\tau) \coloneqq \sum_{j=1}^{m-1} \tfrac{\lambda_j}{p_{j+1}-p_j} \chi_{I_j}(K_\tau)\ .$$

We divided the parameters by the respective interval lengths in this definition such that the parameters $\lambda_j$ correspond to the number of equivalent epitopes in the intervals $I_j$. This can be seen as follows:

When we apply the approximation of the density function to the Fredholm accumulation model, we obtain

$$x \approx \int_0^\infty g(K_\tau)(1-e^{-\frac{a}{K_\tau}})\ dK_\tau \approx \int_0^\infty \sum_{j=1}^{m-1} \frac{\lambda_j}{p_{j+1}-p_j} \chi_{I_j}(K_\tau)(1-e^{-\frac{a}{K_\tau}})\ dK_\tau$$
$$= \sum_{j=1}^{m-1} \frac{\lambda_j}{p_{j+1}-p_j} \int_{I_j} 1-e^{-\frac{a}{K_\tau}}\ dK_\tau \ .$$

Next, assuming the intervals $I_j$ to be sufficiently small, we can approximate the integrals as products. Let $\langle p_j \rangle := \frac{p_{j+1}-p_j}{2}$ denote the midpoint, then

$$\int_{I_j} 1-e^{-\frac{a}{K_\tau}}\ dK_\tau \approx \left(1-e^{-\frac{a}{\langle p_j\rangle}}\right)(p_{j+1}-p_j) \ .$$

Thus, the Fredholm accumulation model becomes

$$x \approx \sum_{j=1}^{m-1} \frac{\lambda_j}{p_{j+1}-p_j} \int_{I_j} 1-e^{-\frac{a}{K_\tau}}\ dK_\tau \approx \sum_{j=1}^{m-1} \frac{\lambda_j}{p_{j+1}-p_j} \left(1-e^{-\frac{a}{\langle p_j\rangle}}\right)(p_{j+1}-p_j)$$
$$= \sum_{j=1}^{m-1} \frac{\lambda_j}{p_{j+1}-p_j}(p_{j+1}-p_j)\left(1-e^{-\frac{a}{\langle p_j\rangle}}\right) = \sum_{j=1}^{m-1} \lambda_j \left(1-e^{-\frac{a}{\langle p_j\rangle}}\right) \ .$$

Comparing the last term with the discrete accumulation model, we can observe that $\{(\lambda_j, \langle p_j\rangle)\}_{j=1}^{m-1}$ defines the epitope classes for the discrete accumulation model. In other words, $\lambda_j$ corresponds to the number of epitopes with $K_\tau = \langle p_j \rangle$. That is, by construction, the number of epitopes with $K_\tau \in I_j$.

## 2.2 Visualization and dose-response contribution

As stated in the main part of the paper and in the methods section, the bin heights are rescaled with respect to the bin widths as they appear in the logarithmically scaled plot. This is a mere visualization aid which ensures that the visual area of peaks in the logarithmically scaled histograms corresponds 1 : 1 to the effect on the dose-response curve. This can easily be illustrated by specifying a density function with two peaks, as the corresponding dose-response curves and peak contributions can readily be obtained from the accumulation model.

For the illustration, consider the sum of two normal distributions $\mathcal{N}(10^{-6}, 3\cdot 10^{-7})$ and $\mathcal{N}(10^{-5}, 3\cdot 10^{-6})$:

$$g(K) = \frac{1}{3\cdot 10^{-7}\cdot\sqrt{2\pi}}\ e^{-\frac{1}{2}\left(\frac{K-10^{-6}}{3\cdot 10^{-7}}\right)^2} + \frac{1}{3\cdot 10^{-6}\cdot\sqrt{2\pi}}\ e^{-\frac{1}{2}\left(\frac{K-10^{-5}}{3\cdot 10^{-6}}\right)^2} \ .$$

The corresponding density function is plotted on a logarithmic scale in Figure 8. Now, this density function can be approximated with a grid. Since the grids are refined

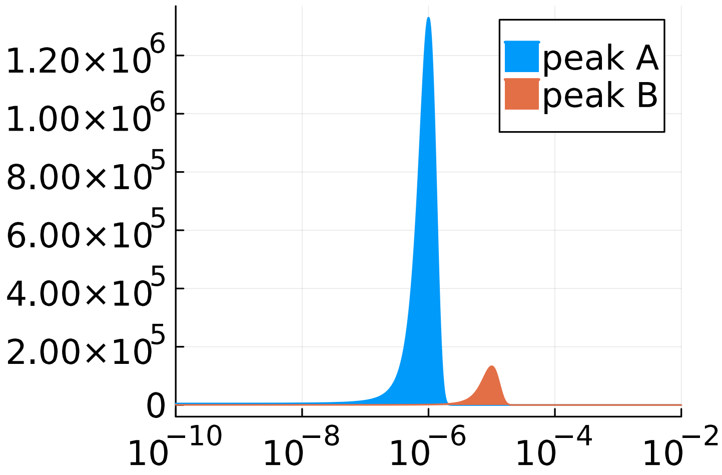


**Supplementary Figure 8**: Plot of $g(K)$.

adaptively during the fitting process, leading to unequal interval sizes, we should do the same for this illustration. Here, we use 25 logarithmically spaced discretization points from $10^{-10}$ to $10^{-6}$, 50 logarithmically spaced discretization points from $10^{-6}$ to $10^{-5}$ and finally 100 logarithmically spaced discretization points from $10^{-5}$ to $10^{-2}$.

Figure 9 shows 3 normalizations for the bin heights: the raw parameters without normalization ($\lambda_i$) in Figure 9a, the parameters divided by the true interval lengths ($^{\lambda_i}/_{V_i}$) in Figure 9b and the parameters divided by the visual interval lengths in the logarithmically scaled plot ($^{\lambda_i}/_{w_i}$) in Figure 9c. Furthermore, the dose-response-curve contributions of the two peaks are plotted in Figure 9d.

The parameters correspond to the number of epitopes with $K_\tau$ in the respective intervals. Thus, without normalization, the bin heights would correspond to the contribution of each bin to the dose-response curve. However, since the bins have different widths, it is difficult to see how many bins comprise a peak. Instead, one is drawn to look at the total surface area of a peak. But this does not correspond to the effect of the peak on the dose-response curve. Hence, not normalizing the bin heights produces a misleading histogram, which is prone to misinterpretation.

Observe that when the bin heights are divided by the true lengths of the intervals, the true shape of the density function $g(K)$ is recovered. Yet, as will be argued in Subsection 3.4, the density function $g(K)$ is of little interest. The density function is meaningless (unit dependent) outside the integral. Only the histogram, resulting from the bin-wise evaluation of the integral, corresponds to the dose-response contribution. In this case, observe that peak B is much smaller than peak A. But the dose-response plot (Fig. 9d) shows that both peaks contribute equally strongly to the overall dose-response curve. Thus, using the interval length normalization for the histogram is also misleading/unintuitive.

As mentioned earlier, one is inclined to interpret the visual surface area of a peak as a measure of the contribution of this peak to the dose-response curve. Dividing the

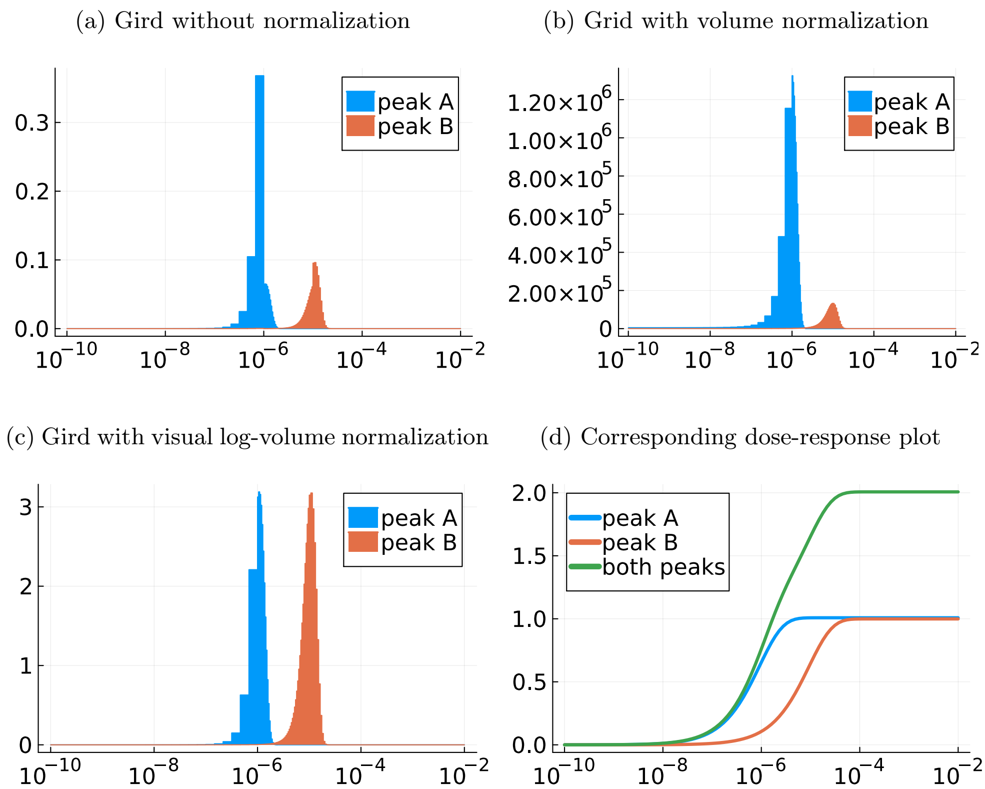


**Supplementary Figure 9**: Comparison of bar-height normalizations for the accessibility histogram.

parameters by the visual bin widths leads to histogram bars whose area is equal to the respective parameters. This is because the visual surface of these rescaled bars is given by $w_i \cdot {}^{\lambda_i}/_{w_i} = \lambda_i$. That the visual area of peaks for this normalization corresponds $1:1$ to the effect on the dose-response curve can be observed in Figure 9c. Both peaks have the same surface area, in accordance with the fact that both peaks contribute equally strongly to the overall dose-response curve (Fig. 9d).

# 3 The accessibility histogram does not depend on the choice of units

A common problem in biological research is that most quantities cannot be measured directly. In these cases, proportional quantities need to be used for the measurement, where the corresponding proportionality constants remain unknown for the most part. This requires additional caution when binding models are used, as a lack of knowledge of these proportionality factors could lead to misinterpretations. Fortunately, the

accumulation model and the shape of the accessibility histogram do not depend on the choice of units in certain circumstances.

### 3.1 Measurement limitations and unknown proportionality factors

Both the antibody concentration $a$ and the concentration of bound antibody-epitope complexes $x$ cannot be set up/measured directly in most biological laboratories. Instead of the antibody concentration $a$ the antibody dilution quotient $\mathfrak{a}$ is used. And instead of the bound antibody-epitope complex concentration $x$ the fluorescence response $\mathfrak{x}$ is measured. These quantities are related by proportionality factors:

$$\mathfrak{a} = \eta a \qquad \text{and} \qquad \mathfrak{x} = \xi x \ .$$

**Remark 1 (Proportionality and Units).**
Measuring a quantity indirectly by measuring a proportional quantity is equivalent to choosing a different measurement unit. For that reason, we will use "proportionality factor" and "unit conversion factor" synonymously in the following.

The conversion between dilution quotient and concentration is quite simple if the concentration of the base dilution is known. And often antibody vendors provide the concentration, albeit in terms of protein weight per volume. However, the ratio of antibodies that have denatured while storing the antibody (freeze/thaw damage) is not known without proper measurements. However, these measurements are either tedious or require special instruments [1] [2]. Hence, they are not performed regularly for simple immunostaining experiments such that $\eta$ is usually not known exactly.

Determining the exact concentration of bound antibody-epitope complexes is similarly difficult with a fluorescence microscope setup. The fluorescence signal, measured by an image sensor, is only proportional to the actual concentration of antibody-epitope complexes. To estimate the actual amount of fluorescence molecules, one can either compare the measured signal intensity with a standardized sample or analyze the bleaching behavior [3][4][5]. However, these approaches require additional steps that do not seem to be done regularly. Thus, also $\xi$ remains unknown in most cases.

### 3.2 Unit conversion factors cancel out in the discrete accumulation model

Note that some properties of the units were already chosen in the derivation of the accumulation model. The concentration of bound antibody-epitope complexes $x$ and the concentration of epitopes $g$ have the same unit. In addition, the antibody concentration $a$ and the accessibility constant $K_\tau$ have the same unit. By restricting the choice of units in this way, we ensured that unit conversion factors cancel out in the accumulation model.

**Corollary 1.**
*Let* $\mathfrak{a} = \eta a$*,* $\mathfrak{K}_{\tau,i} = \eta K_{\tau,i}$*,* $\mathfrak{x} = \xi x$ *and* $\mathfrak{g}_i = \xi g_i$ *be proportional quantities. Furthermore, let*

$$x = \sum_{i=1}^{n} g_i \left(1 - \exp\left(-\frac{a}{K_{\tau,i}}\right)\right) \ .$$

*Then it holds for all proportionality constants* $\eta, \xi > 0$ *and all numbers of epitope classes* $n \in \mathbb{N}$ *that*

$$\mathfrak{x} = \sum_{i=1}^{n} \mathfrak{g}_i \left(1 - \exp\left(-\frac{\mathfrak{a}}{\mathfrak{K}_{\tau,i}}\right)\right) \ .$$

*Proof.*

$$\mathfrak{x} = \xi x = \xi \sum_{i=1}^{n} g_i \left(1 - \exp\left(-\frac{a}{K_{\tau,i}}\right)\right) = \sum_{i=1}^{n} \xi g_i \left(1 - \exp\left(-\frac{\eta a}{\eta K_{\tau,i}}\right)\right)$$

$$= \sum_{i=1}^{n} \mathfrak{g}_i \left(1 - \exp\left(-\frac{\mathfrak{a}}{\mathfrak{K}_{\tau,i}}\right)\right) \ .$$

□

In particular, this corollary implies that the unit conversion factors $\eta$ and $\xi$ need not be known for the discrete accumulation model. At least if it suffices to know/use $\mathfrak{K}_{\tau,i}$ and $\mathfrak{g}_i$ in the same units as $\mathfrak{a}$ and $\mathfrak{x}$ respectively. Note that this is the case for the applications of the accessibility histogram that we presented in this paper.

## 3.3 Conversion factors do not cancel out for the antibody depletion model

Using $x - \beta a$ to model antibody depletion prevents the proportionality factors from canceling out. This can already be seen in the case where only a single epitope class is considered. Yet, although the antibody depletion model can be solved analytically for a single epitope class (cf. preprint https://arxiv.org/abs/2409.06895), it is easier to consider the differential equation.

Let us first consider again the depletion-free accumulation model:

$$\frac{d}{dt}x(t) = k_a a(g - x(t)) = \frac{\tau}{K_\tau}\, a(g - x(t)) \ .$$

Using the proportional quantities, we see that the unit conversion factors cancel out as before:

**Corollary 2.**
*Let* $\mathfrak{a} = \eta a$*,* $\mathfrak{K}_\tau = \eta K_\tau$*,* $\mathfrak{x}(t) = \xi x(t)$ *and* $\mathfrak{g} = \xi g$ *be proportional quantities.*

*Furthermore, let*

$$\frac{d}{dt}x(t) = \frac{\tau}{K_\tau}a(g - x(t)) \ .$$

*Then it holds for all proportionality constants* $\eta, \xi > 0$ *that*

$$\frac{d}{dt}\mathfrak{x}(t) = \frac{\tau}{\mathfrak{K}_\tau}\mathfrak{a}(\mathfrak{g} - \mathfrak{x}(t)) \ .$$

*Proof.*

$$\frac{d}{dt}\mathfrak{x}(t) = \frac{d}{dt}\xi x(t) = \xi\frac{\tau}{K_\tau}a(g - x(t)) = \frac{\tau a}{K_\tau}\xi(g - x(t)) = \frac{\tau\eta a}{\eta K_\tau}\left(\xi g - \xi x(t)\right)$$
$$= \frac{\tau}{\mathfrak{K}_\tau}\mathfrak{a}(\mathfrak{g} - \mathfrak{x}(t)) \ .$$

□

The antibody depletion model is given by the differential equation

$$\frac{d}{dt}x(t) = k_a\left(a - \beta x(t)\right)\left(g - x(t)\right) = \frac{\tau}{K_\tau}\left(a - \beta x(t)\right)\left(g - x(t)\right) \ .$$

If we now consider the proportional quantities, it follows that:

**Corollary 3.**
*Let* $\mathfrak{a} = \eta a$, $\mathfrak{K}_\tau = \eta K_\tau$, $\mathfrak{x}(t) = \xi x(t)$ *and* $\mathfrak{g} = \xi g$ *be proportional quantities. Furthermore, let*

$$\frac{d}{dt}x(t) = \frac{\tau}{K_\tau}\left(a - \beta x(t)\right)\left(g - x(t)\right) \ .$$

*Then it holds for all proportionality constants* $\eta, \xi > 0$ *that*

$$\frac{d}{dt}\mathfrak{x}(t) = \frac{\tau}{\mathfrak{K}_\tau}\left(\mathfrak{a} - \beta\frac{\eta}{\xi}\mathfrak{x}(t)\right)\left(\mathfrak{g} - \mathfrak{x}(t)\right) \ .$$

*Proof.*

$$\frac{d}{dt}\mathfrak{x}(t) = \frac{d}{dt}\xi x(t) = \xi\frac{\tau}{K_\tau}\left(a - \beta x(t)\right)\left(g - x(t)\right) = \frac{\tau(a - \beta x(t))}{K_\tau}\xi(g - x(t))$$
$$= \frac{\tau\eta(a - \beta\frac{\xi}{\xi}x(t))}{\eta K_\tau}(\xi g - \xi x(t)) = \frac{\tau(\eta a - \beta\eta\frac{\xi}{\xi}x(t))}{\eta K_\tau}(\xi g - \xi x(t))$$
$$= \frac{\tau}{\mathfrak{K}_\tau}\left(\mathfrak{a} - \beta\frac{\eta}{\xi}\mathfrak{x}(t)\right)\left(\mathfrak{g} - \mathfrak{x}(t)\right) \ .$$

□

As corollary 3 shows, the unit conversion factors appear explicitly in the equation when units are changed.

## 3.4 Choice of units and the accessibility histogram

So far, we have only considered the discrete accumulation model. Since the Fredholm accumulation model is obtained as an approximation of the discrete accumulation model, one might expect that the unit conversion factors should still cancel out. The intuition is that until the approximation is applied, the discrete accumulation model does not depend on the chosen units. Thus, the approximation as integral should also not depend on the units. Unfortunately, integration by substitution leads to the following result:

**Corollary 4.**
*Let* $\mathfrak{a} = \eta a$, $\mathfrak{K}_\tau = \eta K_\tau$ *and* $\mathfrak{x} = \xi x$ *be proportional quantities. Furthermore, let*

$$x = \int_0^\infty g(K_\tau)\left(1 - \exp\left(-\frac{a}{K_\tau}\right)\right)\, dK_\tau \ .$$

*Then it holds for all proportionality constants* $\eta, \xi > 0$ *that*

$$\mathfrak{x} = \int_0^\infty \mathfrak{g}(\mathfrak{K}_\tau)\left(1 - \exp\left(-\frac{\mathfrak{a}}{\mathfrak{K}_\tau}\right)\right) d\mathfrak{K}_\tau \qquad \textit{where} \qquad \mathfrak{g}(\bullet) = \tfrac{1}{\eta}\xi g(\tfrac{1}{\eta}\bullet) \ .$$

*Proof.*

$$\begin{aligned}
\mathfrak{x} = \xi x = \xi \int_0^\infty g(K_\tau)\left(1 - \exp\left(-\frac{a}{K_\tau}\right)\right) dK_\tau \\
= \int_0^\infty \xi g(K_\tau)\left(1 - \exp\left(-\frac{a}{K_\tau}\right)\right) dK_\tau \\
= \int_{0\eta}^{\infty\eta} \xi g(\tfrac{1}{\eta}\mathfrak{K}_\tau)\left(1 - \exp\left(-\frac{a}{\frac{1}{\eta}\mathfrak{K}_\tau}\right)\right) d(\tfrac{1}{\eta}\mathfrak{K}_\tau) \\
= \int_0^\infty \xi\tfrac{1}{\eta} g(\tfrac{1}{\eta}\mathfrak{K}_\tau)\left(1 - \exp\left(-\frac{\mathfrak{a}}{\mathfrak{K}_\tau}\right)\right) d\mathfrak{K}_\tau \\
=: \int_0^\infty \mathfrak{g}(\mathfrak{K}_\tau)\left(1 - \exp\left(-\frac{\mathfrak{a}}{\mathfrak{K}_\tau}\right)\right) d\mathfrak{K}_\tau \ .
\end{aligned}$$

□

As was the case for the discrete accumulation model, a coefficient $\xi$ converts the values of $g(\bullet)$ to the same unit in which $\mathfrak{x}$ is measured. However, the transformation $\mathfrak{g}(\bullet) = \frac{1}{\eta}\xi g(\frac{1}{\eta}\bullet)$ also contains the unit conversion factor $\eta$. Thus, the density function $g(\bullet)$ depends on the unit of the antibody concentration. This may seem unsettling at

first, as it could mean that the accessibility histogram, which is derived from the density function $g(\bullet)$, depends on the chosen antibody concentration unit. Furthermore, the additional coefficient $\frac{1}{\eta}$ seems unnatural as it converts the values of the density function $g(\bullet)$ with respect to the conversion factor for the antibody concentration (which should not have an effect on epitope measurement).

Before we address the implications for the accessibility histogram, let us recall how the integral equation came about. It was a mere approximation of a discrete sum, to ease the applicability of the model. In fact, to retrieve the discrete model, the density function $g(\bullet)$ must be a sum of delta-functions, which only makes sense when integrated. In that regard, the density function $g(\bullet)$ may be understood in the same way as a probability density function. It is just a density function whose meaning arises only after integration. For probability density functions, integration over a subset yields the probability that the value of the random variable is in the subset. Here, integration over a subset leads to the response contribution of epitopes with $K_\tau$ in the subset. Thus, any odd behavior of the density function $g(\bullet)$ upon unit changes can be neglected, as long as the integral remains unchanged. Fortunately, this is the case, as can be seen in proof 3.4.

The fact that the integral does not depend on the choice of units also addresses concerns about the accessibility histogram. After all, the accessibility histogram is obtained by evaluating the integral piecewise over the bins (i.e. intervals) of the histogram. For this, the density function $g(\bullet)$ is assumed to be constant over the bins (cf. Methods "adaptive grids" and Section 2):

$$g(\bullet) = \sum_{j=1}^{m} \tfrac{f_j}{\mathrm{Vol}(I_j)} \chi_{I_j}(\bullet) \qquad \text{where} \qquad \chi_{I_j}(\bullet) = \begin{cases} 1 & ,\bullet \in I_j \\ 0 & ,\text{else} \end{cases}$$

$$\Rightarrow \quad x = \int_0^\infty g(K_\tau)(1-\exp(-\tfrac{a}{K_\tau}))\, dK_\tau = \sum_{j=1}^{m} \tfrac{f_j}{\mathrm{Vol}(I_j)} \int_{I_j} (1-\exp(-\tfrac{a}{K_\tau}))\, dK_\tau \ .$$

Thus, to investigate the effect of the choice of units, we should focus on decompositions of the density function $g(\bullet)$:

**Theorem 1.**
*Let $I \subseteq \mathbb{R}_{>0}$ be a right-open interval and let $I_j = [p_j, q_j)$ be intervals with $q_j > p_j$ and $p_{j+1} = q_j$ such that $\bigcup_{j=1}^m I_j = I$. Furthermore, consider the following decomposition of the density function:*

$$g(K_\tau) = \sum_{j=1}^{m} f_j \tfrac{1}{q_j - p_j} \chi_{[p_j, q_j)}(K_\tau) \ .$$

*In addition, consider the following integral and its approximation*

$$H_j := \int_{p_j}^{q_j} \tfrac{1}{q_j - p_j} (1-\exp(-\tfrac{a}{K_\tau}))\, dK_\tau \qquad \text{and} \qquad h_j := 1-\exp\left(-\frac{a}{q_j - p_j}\right) .$$

*Then the Fredholm accumulation model (over I) and its approximation read:*

$$x = \sum_{j=1}^{m} f_j H_j \qquad \textit{and} \qquad x_{approx.} := \sum_{j=1}^{m} f_j h_j \ .$$

*Let* $\mathfrak{x} = \xi x$, $\mathfrak{f}_j = \xi f_j$ *and* $\mathfrak{a} = \eta a$, $\mathfrak{K}_\tau = \eta K_\tau$, $\mathfrak{p}_j = \eta p_j$, $\mathfrak{q}_j = \eta q_j$ *be proportional quantities, then it holds for all* $\xi, \eta > 0$ *that*

$$\mathfrak{x} = \sum_{j=1}^{m} \mathfrak{f}_j H_j \ , \qquad \mathfrak{x}_{approx.} = \sum_{j=1}^{m} \mathfrak{f}_j h_j \ ,$$

$$H_j = \int_{\mathfrak{p}_j}^{\mathfrak{q}_j} \frac{1}{\mathfrak{q}_j - \mathfrak{p}_j} \left(1 - \exp\left(-\frac{\mathfrak{a}}{\mathfrak{K}_\tau}\right)\right) \, d\mathfrak{K}_\tau \ , \qquad h_j = 1 - \exp\left(-\frac{\mathfrak{a}}{\mathfrak{q}_j - \mathfrak{p}_j}\right) \ .$$

*Proof.* The term $H_j$ is defined such that the expression $x = \sum_{j=1}^{m} f_j H_j$ is true:

$$\begin{aligned} x &= \int_0^\infty g(K_\tau)(1 - \exp(-\tfrac{a}{K_\tau})) \, dk \\ &= \int_0^\infty \sum_{j=1}^{m} f_j \tfrac{1}{q_j - p_j} \chi_{[p_j, q_j)}(K)(1 - \exp(-\tfrac{a}{K_\tau})) \, dK_\tau \\ &= \sum_{j=1}^{m} f_j \int_0^\infty \chi_{[p_j, q_j)}(K) \tfrac{1}{q_j - p_j}(1 - \exp(-\tfrac{a}{K_\tau})) \, dK_\tau \\ &= \sum_{j=1}^{m} f_j \int_{p_j}^{q_j} \tfrac{1}{q_j - p_j}(1 - \exp(-\tfrac{a}{K_\tau})) \, dK_\tau = \sum_{j=1}^{m} f_j H_j \ . \end{aligned}$$

Furthermore, $x_{\text{approx.}} = \sum_{j=1}^{m} f_j h_j$ is just a definition. Thus, it remains to show the properties for the proportional quantities.

First, observe that the proportionality factor simply cancels out in $h_j$:

$$1 - \exp\left(-\frac{\mathfrak{a}}{\mathfrak{q}_j - \mathfrak{p}_j}\right) = 1 - \exp\left(-\frac{\eta a}{\eta q_j - \eta p_j}\right) = 1 - \exp\left(-\frac{a}{q_j - p_j}\right) = h_j \ .$$

For $H_j$ we use integration by substitution:

$$\begin{aligned}
H_j &= \int_{p_j}^{q_j} \frac{1}{q_j - p_j} \left(1 - \exp\left(-\frac{a}{K_\tau}\right)\right) \, dK_\tau \\
&= \int_{\eta p_j}^{\eta q_j} \frac{1}{q_j - p_j} \left(1 - \exp\left(-\frac{a}{\frac{1}{\eta}\mathfrak{K}_\tau}\right)\right) \, d(\tfrac{1}{\eta}\mathfrak{K}_\tau) \\
&= \int_{\eta p_j}^{\eta q_j} \frac{1}{\eta} \cdot \frac{1}{(q_j - p_j)} \left(1 - \exp\left(-\frac{\eta a}{\mathfrak{K}_\tau}\right)\right) \, d\mathfrak{K}_\tau \\
&= \int_{\eta p_j}^{\eta q_j} \frac{1}{\eta q_j - \eta p_j} \left(1 - \exp\left(-\frac{\eta a}{\mathfrak{K}_\tau}\right)\right) \, d\mathfrak{K}_\tau \\
&= \int_{\mathfrak{p}_j}^{\mathfrak{q}_j} \frac{1}{\mathfrak{q}_j - \mathfrak{p}_j} \left(1 - \exp\left(-\frac{\mathfrak{a}}{\mathfrak{K}_\tau}\right)\right) \, d\mathfrak{K}_\tau \ .
\end{aligned}$$

Thus, $H_j$ and $h_j$ do not depend on the proportionality factor $\eta$. Furthermore, they do not depend on $\xi$, as no quantity used to calculate them depends on $\xi$. Thus, calculating $\mathfrak{x}$ and $\mathfrak{x}_{\text{approx.}}$ becomes a trivial insertion of the definitions $\mathfrak{x} = \xi x$ and $\mathfrak{f}_j = \xi f_j$:

$$\mathfrak{x} = \xi x = \xi \sum_{j=1}^{m} f_j H_j = \sum_{j=1}^{m} \xi f_j H_j = \sum_{j=1}^{m} \mathfrak{f}_j H_j \ ,$$

$$\mathfrak{x}_{\text{approx.}} = \xi x_{\text{approx.}} = \xi \sum_{j=1}^{m} f_j h_j = \sum_{j=1}^{m} \xi f_j h_j = \sum_{j=1}^{m} \mathfrak{f}_j h_j \ .$$

□

**Remark 2 (Interpretation of theorem 1).**
Essentially, Theorem 1 has the following meaning: When the antibody and epitope concentrations are measured in other units than number of particles per volume/surface area, only the numbers written on the axes of the accessibility histogram change. The shape of the histogram remains unchanged. That is, the relation between the $\{\mathfrak{f}_j\}$ (measured in the new units) is the same as the relation between the $\{f_j\}$. Furthermore, the intervals $[p_j, q_j)$ to which the coefficients $f_j$ belong are transformed (according to the antibody-concentration-unit-change) to $[\mathfrak{p}_j, \mathfrak{q}_j)$, such that the relation between the bin widths of the histogram is preserved.

# 4 Depletion correction

In Subsection 3.3 we have seen that the conversion factor $\beta$ for the antibody depletion term $a - \beta x(t)$ explicitly depends on the choice of units (see Corollary 3). Since the

unit conversion between the measured response signal and the concentration of bound antibodies is unknown in most cases, $\beta$ often remains unknown. For this reason, we have neglected antibody depletion in the accumulation model so far. Nevertheless, even without considering the accumulation model with antibody depletion, we can estimate the worst possible depletion from the data, allowing us to estimate the worst-case error for neglecting antibody depletion.

Let $\{(\mathfrak{a}_i, \mathfrak{x}_i)\}_{i=1}^n$ be dose-response data in experimental units, e.g. dilution quotient and fluorescence signal. We recall the accumulation model: The antibodies bind permanently to the epitopes, so the concentration of bound antibodies increases over time. As the antibodies bind, the concentration of unbound antibodies decreases. After the incubation phase, the number of unbound antibodies has decreased exactly by the number of bound antibodies. In terms of dilution quotient $\mathfrak{a}$ and fluorescence signal $\mathfrak{x}$, the resulting dilution quotient of unbound antibodies $\mathfrak{b}$ is given by

$$\mathfrak{b} = \mathfrak{a} - \boldsymbol{\beta}\mathfrak{x} \ ,$$

where we have used the conversion factor $\boldsymbol{\beta}$, that is still unknown. Note, however, that $\boldsymbol{\beta}$ is a fixed constant for a given choice of units. Hence, for all data points the remaining free antibody dilution quotient is

$$\mathfrak{b}_i = \mathfrak{a}_i - \boldsymbol{\beta}\mathfrak{x}_i \ .$$

At this point, we still do not know the conversion factor $\boldsymbol{\beta}$. But, since the remaining antibody dilution quotient must not be negative, we can estimate the largest possible conversion factor

$$\widehat{\boldsymbol{\beta}} \coloneqq \max\{\gamma \geq 0 \mid \mathfrak{a}_i - \gamma\mathfrak{x}_i \geq 0 \quad \forall\ i\} \ .$$

Then, following the idea of [6], we can correct the initial antibody dilution quotients, using the largest possible conversion factor.

$$\widetilde{\mathfrak{b}}_i \coloneqq \mathfrak{a}_i - \widehat{\boldsymbol{\beta}}\mathfrak{x}_i \qquad \rightsquigarrow \qquad \text{corrected data:} \quad \{(\widetilde{\mathfrak{b}}_i, \mathfrak{x}_i)\}_{i=1}^n \ .$$

This constitutes a worst-case scenario for two reasons. First, by definition $\widehat{\boldsymbol{\beta}}$ is larger than the true conversion factor $\boldsymbol{\beta}$, which leads to smaller values for $\widetilde{\mathfrak{b}}_i$. Second, using the values $\widetilde{\mathfrak{b}}_i$ assumes that the antibodies were depleted for the whole antibody incubation phase, when in reality the antibody dilution quotient started from the initial dilution quotient in the beginning, gradually decreasing over time.

Figures 10 and 11 show depletion-corrected dose-response curves (for the data from Figures 1 and 2) and the corresponding accessibility histograms. For the plots, but not for the curve fitting, any data points with corrected dilution quotient equal to zero were removed, as they would conflict with the logarithmic scale.

Observe that the depletion correction only affects the lower dilution-quotient data points, as expected. Nevertheless, the obtained accessibility histograms remain almost the same as for the uncorrected dose-response curves in Figure 2. Only a slight increase in the leftmost peak can be observed in Fig. 10a and 10c.

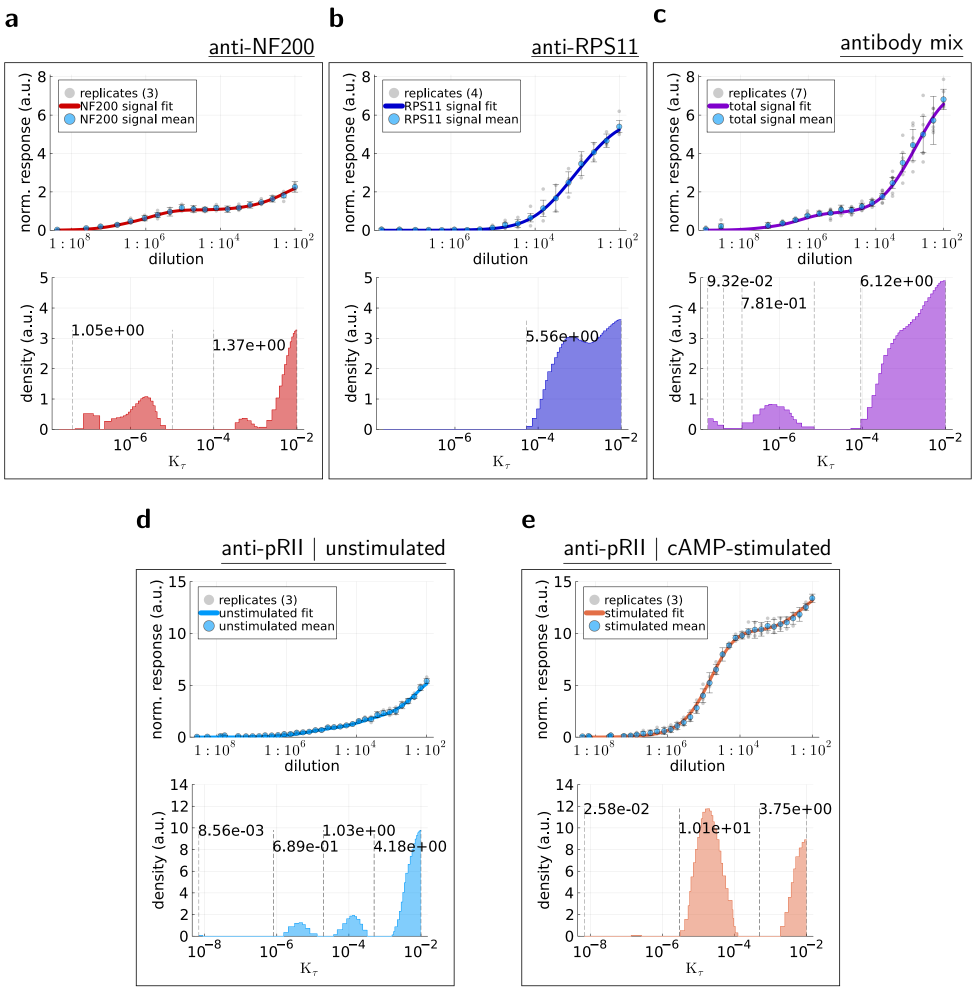


**Supplementary Figure 10**: Depletion-corrected dose-response curves (mean values of replicates ($n$ indicated in legends); error bars = sample std) from Figure 2 and the corresponding accessibility histograms.

For the incubation-time experiments, only the histogram for the 21:20 h incubation (Figure 11c) is meaningfully affected by the depletion correction. The strong leftward-shift of the low-$K_\tau$ peaks (order of magnitude lower $K_\tau$ values) indicates substantial depletion. But more interestingly, the peak dispersion becomes very pronounced, despite using the strong regularization parameter ($\alpha = 500$). This corroborates our interpretations from the main part further. First, the strong depletion for the 21:20 h incubation agrees with the assumption that longer incubation times allow more antibodies to bind (for a given concentration). Second, the peak dispersion, which was only

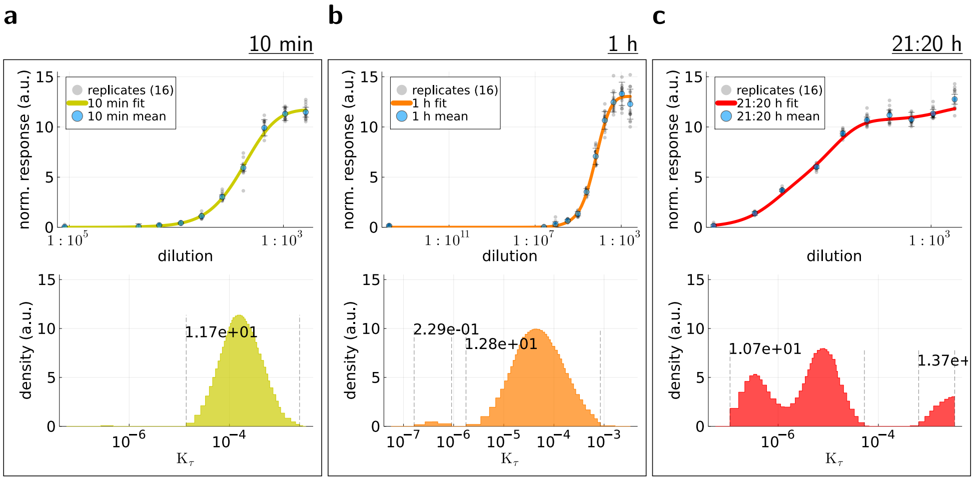


**Supplementary Figure 11**: Depletion-corrected dose-response curves (mean values of $n = 16$ replicates; error bars = sample std) from Figure 1 and the corresponding accessibility histograms.

clearly visible for weaker regularization parameters in the uncorrected data, gets reaffirmed, which indicates that it is not just a fitting artifact for too low regularization parameters.

In summary, depletion effects do not alter the results and conclusions of the main part.

# 5 Anti-pRII in mouse DRG neurons

The anti-pRII experiments from the main part of the paper were also performed with mouse dorsal root ganglion (DRG) neurons.

Since mice had to be sacrificed and the resulting tissue does not only consist of neurons, the wells and thus the microscopy images contained significantly fewer target cells (ranging from 150 to 500 neurons per well), compared to the HeLa experiments of the main part (around 5000 cells per well).

Figures 12 and 13 show the resulting accessibility histograms and peak-analysis plots. However, note that the measurement uncertainties are not the standard deviations of the replicates but $\pm 8$ for each data point (mean of replicates). These "constructed" uncertainties were necessary to improve the fitting results. In addition, $\alpha = 0.5$ was used as regularization parameter, instead of $\alpha = 500$. For further details, see Supplementary Notes 6 and 7.

In summary, the overall result is the same as for HeLa cells. The signal increase is caused solely by an increase in the middle peak, while the rightmost peak remains almost unchanged. However, compared to HeLa cells, the increase in the middle peak is much smaller for DRG neurons (only 2-fold instead of 5-fold). Furthermore, the

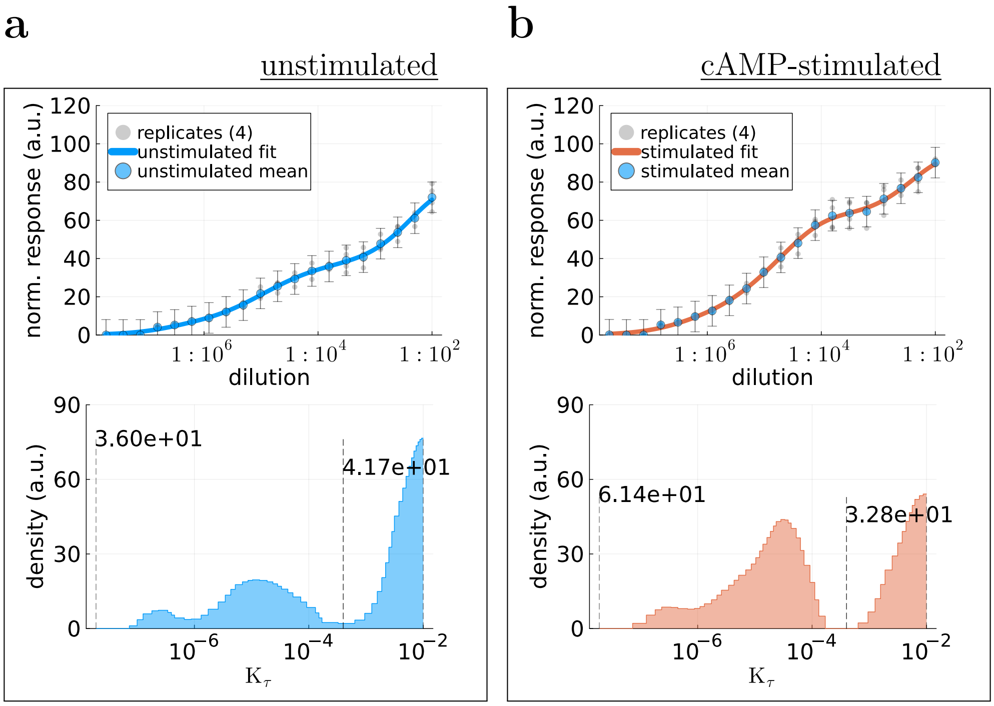


**Supplementary Figure 12**: Dose-response curves (mean values of $n = 4$ replicates; error bars = sample std) and accessibility histograms for the anti-pRII antibody in mouse DRG neurons. Uncertainty and error bars were set to $\pm 8$ to improve the fitting result (cf. Supplementary Note 7)

two low-$K_\tau$ peaks of unstimulated DRG neurons remain visible as distinct peaks for cAMP-stimulated DRG neurons.

The differences of the anti-pRII dose-response behavior in DRG neurons, compared to HeLa cells, may be explained by the amount of PKA present in the cells. Although the zero conditions, which are used to normalize the response values, are not identical (zero control withh unstimulated HeLa cells vs. zero control with unstimulated DRG neurons), the zero-control baselines should not be too different. Thus, the almost 10-times higher response values in DRG neurons, compared to HeLa cells, show that much higher levels of the pRII epitope were present in the DRG neurons. This also means that there were much higher levels of PKA in DRG neurons than in HeLa cells. Since the stimulation procedure was the same for both experimental series, it can be speculated that the applied cAMP concentration sufficed to activate the majority of PKA present in HeLa cells, but only activated a certain proportion of PKA in DRG neurons.

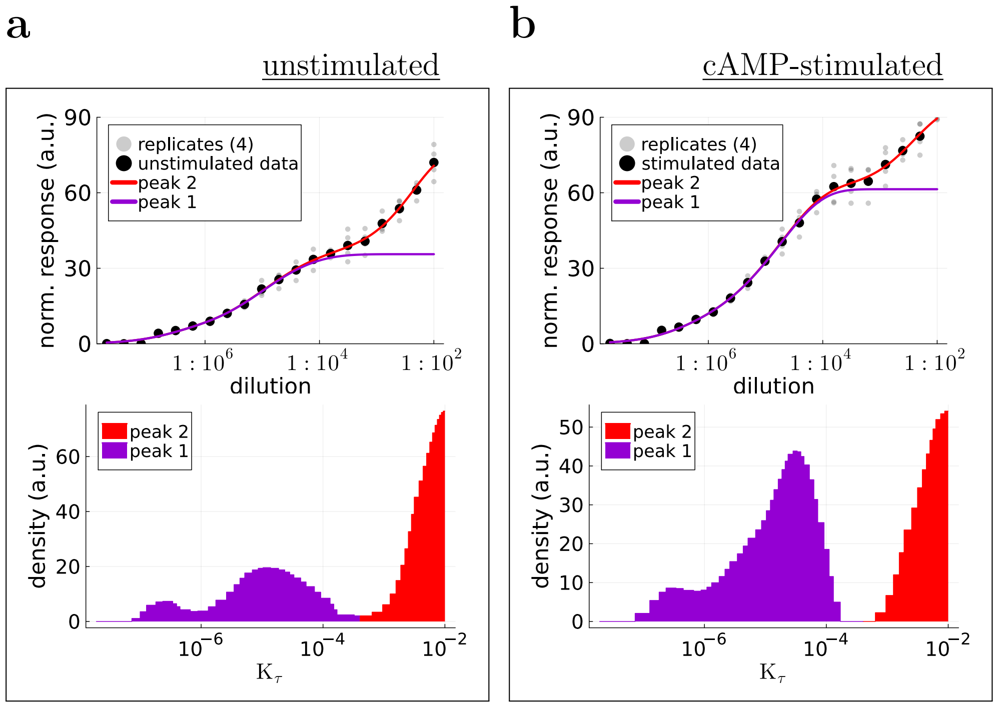


**Supplementary Figure 13**: Analysis of peak contributions for the anti-pRII antibody in mouse DRG neurons.

## 6 Choice of regularization parameter

At the end of the "Antibody accumulation and accessibility histograms" section, it was recommended to consider large and moderate regularization parameters, without additional clarification when a regularization parameter is "large" or "moderate". Furthermore, no objective method was provided to pick the regularization parameter. The simple reason behind this vagueness is that the regularization effect of a given regularization parameter $\alpha$ depends on the dose-response data. To understand this dependence, let us consider the fitting objective function from the Methods section. Fully expanding the terms, it reads

$$\text{obj}(\lambda) = \underbrace{\sum_{i=1}^{n} \frac{(r_i - f(d_i, \lambda))^2}{(2\Delta r_i)^2}}_{=:L(\lambda)} + \underbrace{\frac{\alpha}{m^2} \sum_{j=1}^{m-2} \left( \frac{\lambda_{j+1}}{\log(p_{j+2}) - \log(p_{j+1})} - \frac{\lambda_j}{\log(p_{j+1}) - \log(p_j)} \right)^2}_{=:\alpha S(\lambda)}, \tag{1}$$

where we have defined the functions $L(\lambda)$ and $S(\lambda)$ for easier reference. Since the fully expanded expression is rather unwieldy, let us use the expression

$$\text{obj}(\lambda) = L(\lambda) + \alpha S(\lambda)$$

from here on.

The function $L(\lambda)$ is just the least squares objective function, weighted by the measurement uncertainties. And $S(\lambda)$ acts as smoothing objective function that becomes larger when peaks become sharper in the logarithmically plotted histogram. To determine the optimal model parameter configuration $\widehat{\lambda}$, the objective function is numerically minimized. In this case, the model parameter configuration must describe the data sufficiently well, so that $L(\lambda)$ becomes sufficiently small, but the parameter configuration must not lead to sharp peaks, so that $\alpha S(\lambda)$ becomes sufficiently small. Note that the value of $L(\lambda)$ only depends on the differences between data points and the model curve, irrespective of the histogram shape. And vice versa, the value of $S(\lambda)$ only depends on the histogram shape, irrespective of the shape of the model curve.

In itself, simultaneously minimizing $L(\lambda)$ and $S(\lambda)$ need not be a contradiction. But for data that belongs to sharp peaks, $L(\lambda)$ and $S(\lambda)$ cannot be minimized at the same time. Instead, the optimal model parameter configuration $\widehat{\lambda}$ will be a tradeoff between a model curve that describes the data points well and a histogram that does not have sharp peaks. Since the regularization parameter $\alpha$ determines the weight of the smoothing objective function, the optimal model parameter configuration must be a function of the regularization parameter: $\widehat{\lambda} = \widehat{\lambda}(\alpha)$.

Supplementary Note 8 shows the fitting results of all dose-response curves of the manuscript for different regularization parameters. Disregarding the cases where errors needed to be changed (the fitting process failed, as explained in Supplementary Note 7), the model curve that is closest to the data points is obtained for $\alpha = 0$. Increasing the regularization parameter increases the smoothness of the accessibility histogram, but also increases the discrepancy between the model curve and the data points. This is not just a visual effect, but can also be quantified with the mean squared error

$$\text{MSE} = \frac{1}{n} \sum_{i=1}^{n} \left(r_i - f(d_i, \lambda)\right)^2 \ .$$

However, increasing the regularization parameter $\alpha$ also increases the objective function value $\text{obj} = \text{obj}(\widehat{\lambda}(\alpha))$.

The correlation between mean squared error and regularization parameter is inconspicuous. After all, the mean squared error is closely related to the least squares objective function. But the correlation between objective function value and regularization parameter seems surprising. Yet, upon closer inspection, also the second correlation is reasonable.

The regularization parameter has two effects. First it determines the contribution scale of the smoothing objective function to the overall objective function. In doing so, it directly increases the value of the overall objective function. Second, it enforces

optimal model parameter configurations $\widehat{\lambda}(\alpha)$ that try to minimize $S(\lambda)$, albeit at the cost of increasing the value of $L(\lambda)$.

At this point, one might be tempted to conclude that there is an optimal regularization parameter that balances the different effects. In other words, one might assume that there is an optimal regularization parameter that leads to the smallest objective function value (for a given data set) and that one can approach this optimal regularization parameter in an algorithmic manner. Unfortunately, this is not the case. Since $S(\lambda)$ is positive, the smallest objective function value will always be achieved for $\alpha = 0$ (i.e. no regularization).

Even worse, assuming a continuous dependence of the optimal model parameter configuration $\widehat{\lambda}(\alpha)$ on the regularization parameter $\alpha$ implies that a local maximum must be crossed (for increasing $\alpha$ starting from zero), before a local minimum can exists, since $L(\lambda) \geq 0$ and $S(\lambda) \geq 0$ depend continuously on the parameter configuration. In other words, starting from a small regularization parameter, one will always find $\alpha = 0$ as (locally) optimal regularization parameter. And using the mean squared error as a metric to optimize the regularization parameter will lead to the same result, since the mean squared error is closely related to $L(\lambda)$.

So, with the most natural metrics to determine the quality of the model parameter configuration, the regularization parameter cannot be determined in the form of an optimization problem. It remains to pick the regularization parameter by allowing a certain deviation of the model curve from the data points. This is ultimately a subjective choice. And picking a random threshold, e.g. for the mean squared error, is not more objective than a visual inspection of the plot.

Finally, recommending regularization parameters based on any number of validation experiments is hardly feasible. The least squares objective function $L(\lambda)$ depends on the scale of the response values $r_i$, the measurement uncertainties $\Delta r_i$ and the number of data points, etc. And although the smoothing objective function $S(\lambda)$ does not contain terms involving the data points, it depends on the model parameter configuration, which must describe the data. Overall, the effect of the regularization parameter as a scale for the regularization strength depends non-trivially on the data being investigated.

In the end, one might still be concerned about the subjectivity of "choosing" a regularization parameter. But focusing too much on this subjectivity can distract from the many other subjective choices that are regularly made and that are inevitable for inference problems. For example, what rational is used to determine the number of replicates? More is better, so why stop at any given number? Why does one pick a particular number of intermediate dilution steps? More is better, so why stop at any given number? Why choose a smoothing regularization? Or, expressed in the Bayesian context: Why assume a priori that smooth parameter configurations are more probable than other configurations?

# 7 Data uncertainties and fitting tips

Contrary to previous analyses, where the standard deviation of replicates was used as measurement uncertainty, the analysis of anti-pRII dose-response curves in mouse

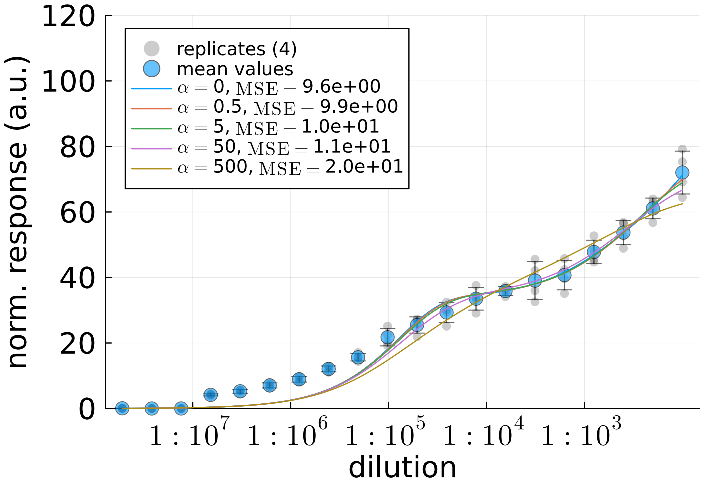


**Supplementary Figure 14**: Comparison of fitting results for different regularization parameters (data: anti-pRII antibody in unstimulated mouse DRG neurons; mean values of $n = 4$ replicates; error bars = sample std).

DRG neurons (Supplementary Note 5) was performed with ±8 as measurement uncertainty for each data point. Defining these artificial uncertainties became necessary because the standard deviations of the data points for the lowest antibody concentrations were too small. Although small measurement errors are usually desirable, they prevent model curves that agree with the first non-zero response values in this case, as can be seen in Figure 14 (see Subsection 8.11 for the corresponding accessibility histograms).

Since the weight (i.e. the importance) of a data point in the least squares objective function $L(\lambda)$ is determined by the measurement uncertainty (cf. Eq. (1)), data points with small measurement uncertainties become more important for the curve fitting. For the dose-response data of Figure 14, the three lowest antibody concentrations are thus far more important than the subsequent data points. And since the curvature (in the logarithmic scale) is limited by the model shape $g_i(1 - e^{-a/K_{\tau,i}})$ (for a single epitope class), not even arbitrarily sharp peaks allow for model curves that can remain flat for the three lowest concentrations and then "jump up" to cover the subsequent data points. In consequence, the model curves miss the data points between $1 : 10^7$ and $1 : 10^5$ for all regularization parameters.

By assigning the same measurement uncertainty of ±8 to each data point, model curves can be obtained that agree with the majority of the data points, as can be seen in Figure 15 (see Subsection 8.9 for the corresponding accessibility histograms). Here, the model curves deviate form the second and third data point (viewed from left to right) to capture the behavior of the subsequent data points.

In summary, when the data uncertainty is smaller than visual noise or jumps in the dose-response curve, uniform uncertainties can be tested to improve the fitting results.

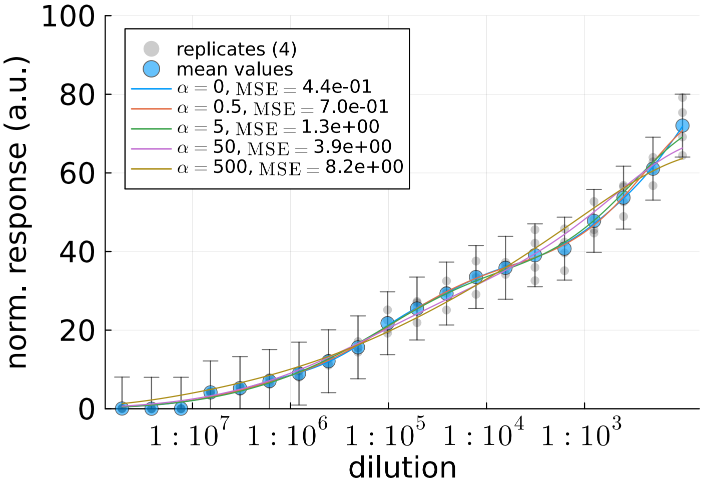


**Supplementary Figure 15**: Comparison of fitting results for different regularization parameters, using $\pm 8$ as measurement uncertainty (data: anti-pRII antibody in unstimulated mouse DRG neurons; mean values of $n = 4$ replicates).

# 8 Additional regularization-parameter plots

The vertical scale is the same for all histogram plots, but was optimized for the regularization parameters $\alpha > 0$. Thus, the peaks for $\alpha = 0$ extend beyond the plot.

## 8.1 Incubation time experiments: 10 min incubation

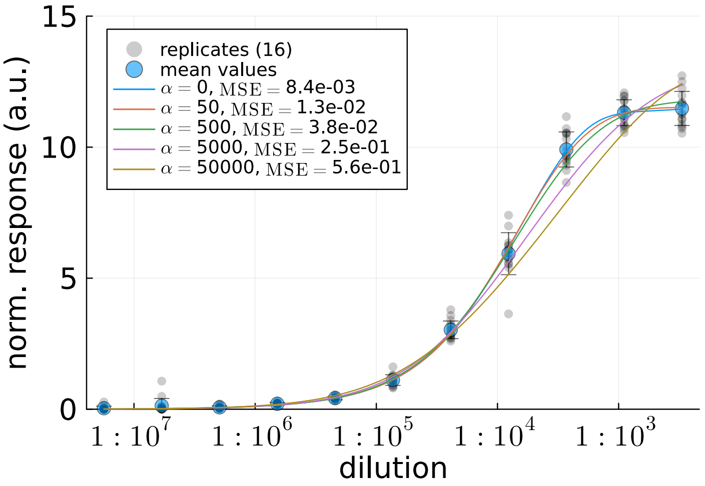


**Supplementary Figure 16**: Comparison of fitting results for different regularization parameters (mean values of $n = 4$ replicates; error bars = sample std).

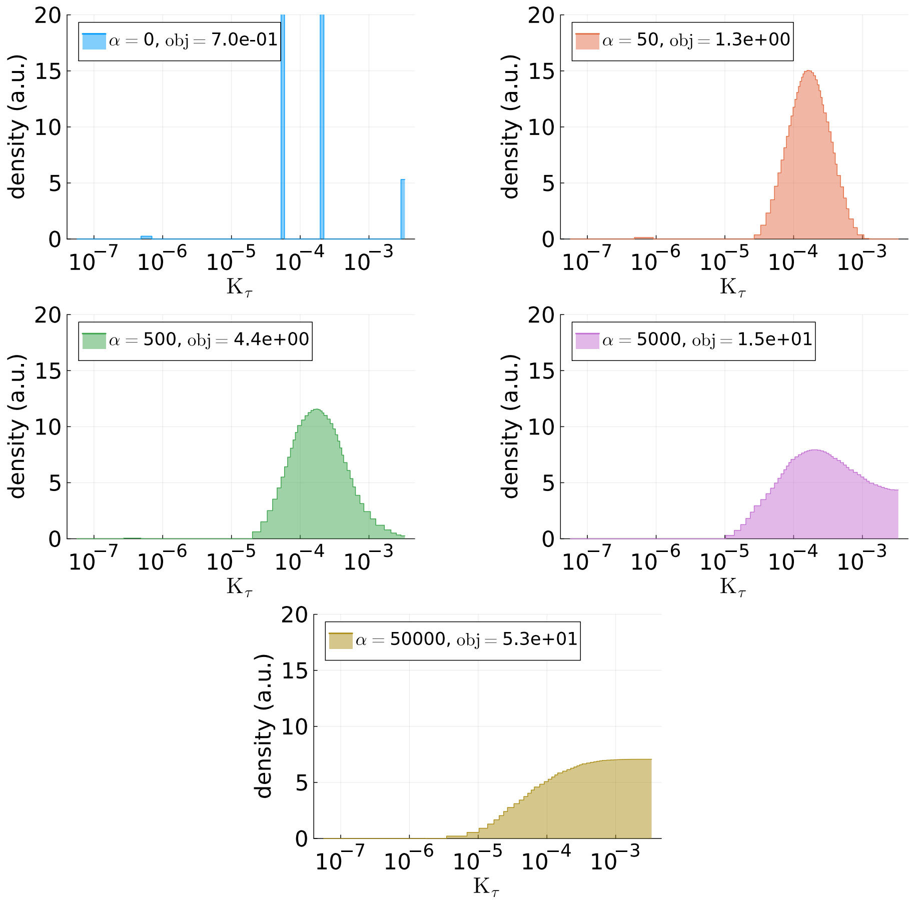


**Supplementary Figure 17**: Color-matched accessibility histograms for the different regularization parameters.

## 8.2 Incubation time experiments: 1 h incubation

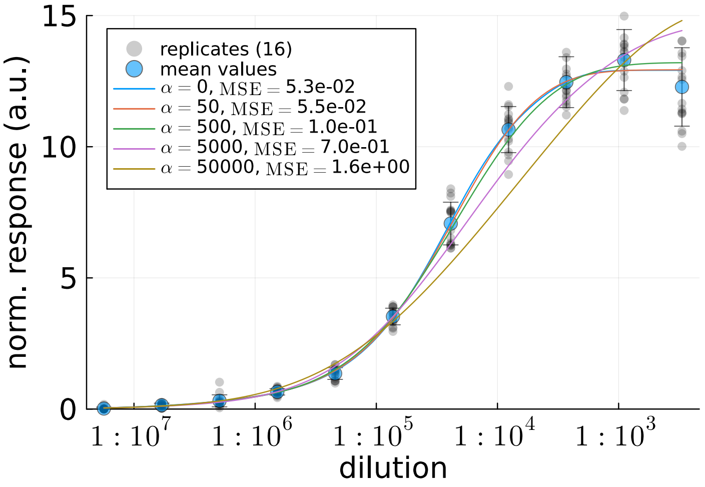


**Supplementary Figure 18**: Comparison of fitting results for different regularization parameters (mean values of $n = 4$ replicates; error bars = sample std).

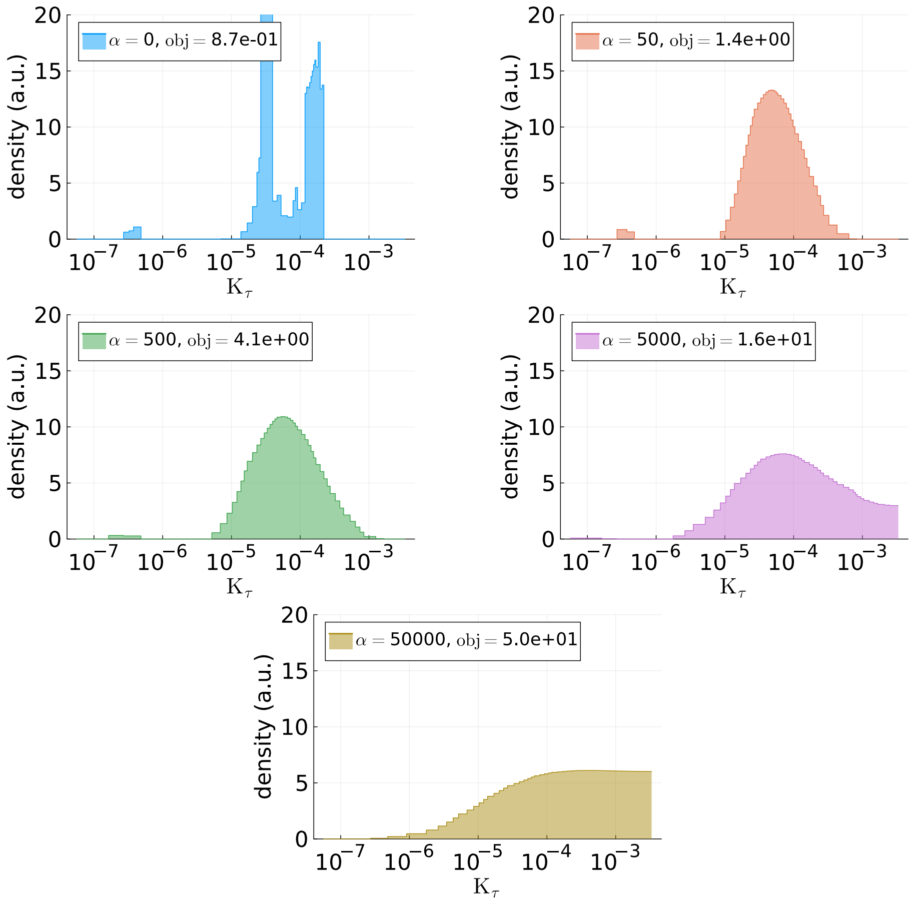


**Supplementary Figure 19**: Color-matched accessibility histograms for the different regularization parameters.

## 8.3 Incubation time experiments: 21:20 h incubation

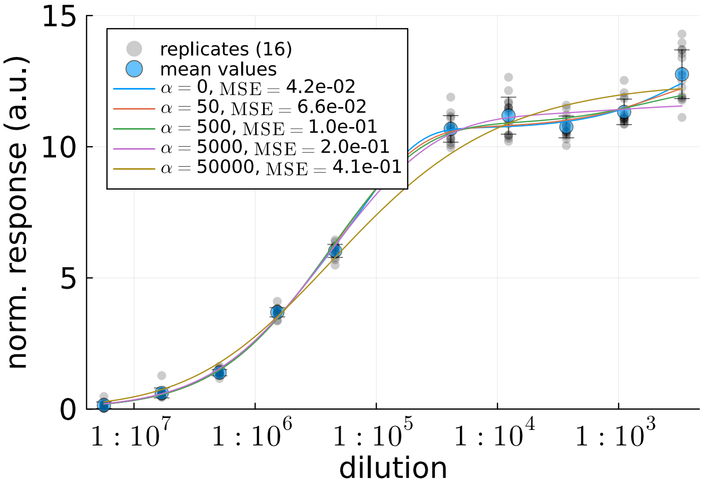


**Supplementary Figure 20**: Comparison of fitting results for different regularization parameters (mean values of $n = 4$ replicates; error bars = sample std).

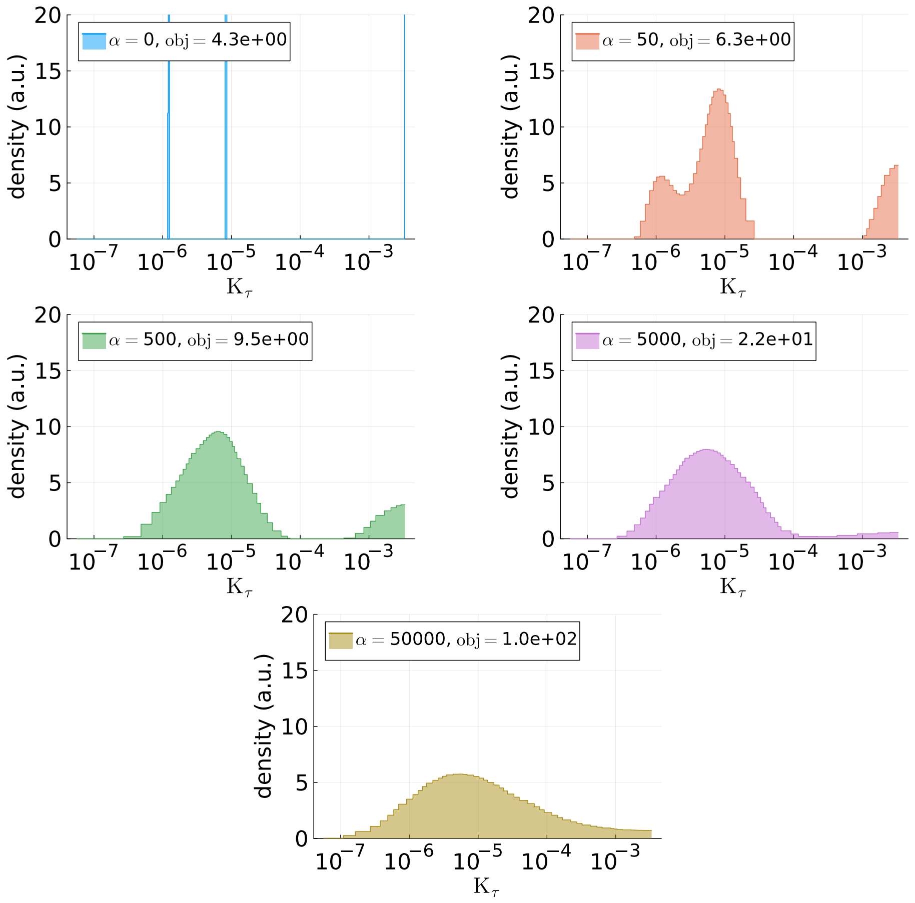


**Supplementary Figure 21**: Color-matched accessibility histograms for the different regularization parameters.

## 8.4 Histogram validation: Anti-Nf200

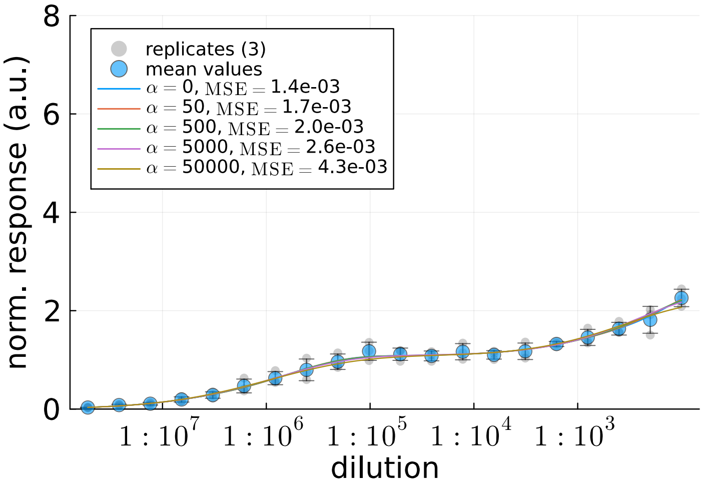


**Supplementary Figure 22**: Comparison of fitting results for different regularization parameters (mean values of $n = 3$ replicates; error bars = sample std).

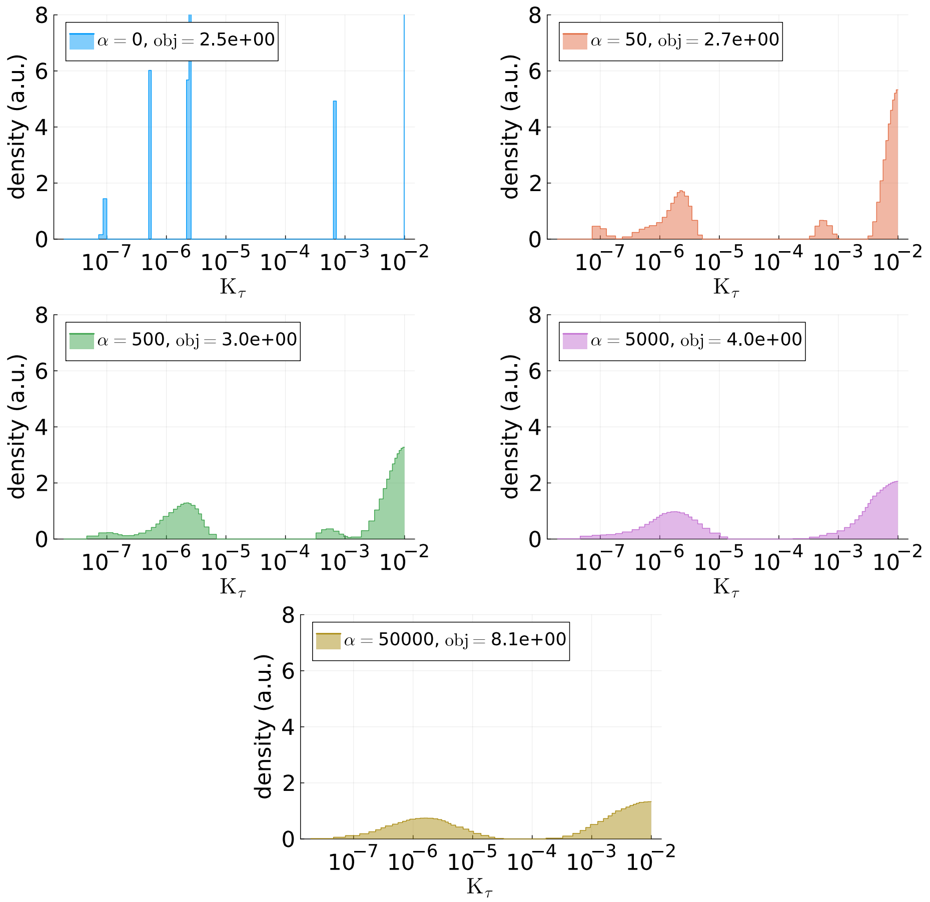


**Supplementary Figure 23**: Color-matched accessibility histograms for the different regularization parameters.

## 8.5 Histogram validation: Anti-RPS11

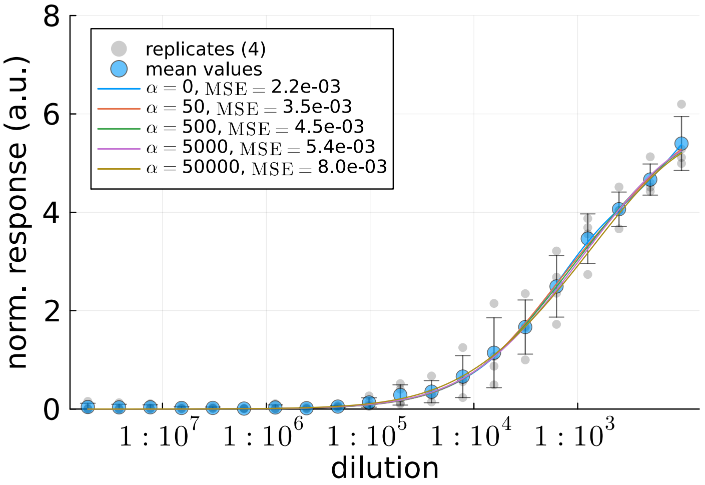


**Supplementary Figure 24**: Comparison of fitting results for different regularization parameters (mean values of $n = 4$ replicates; error bars = sample std).

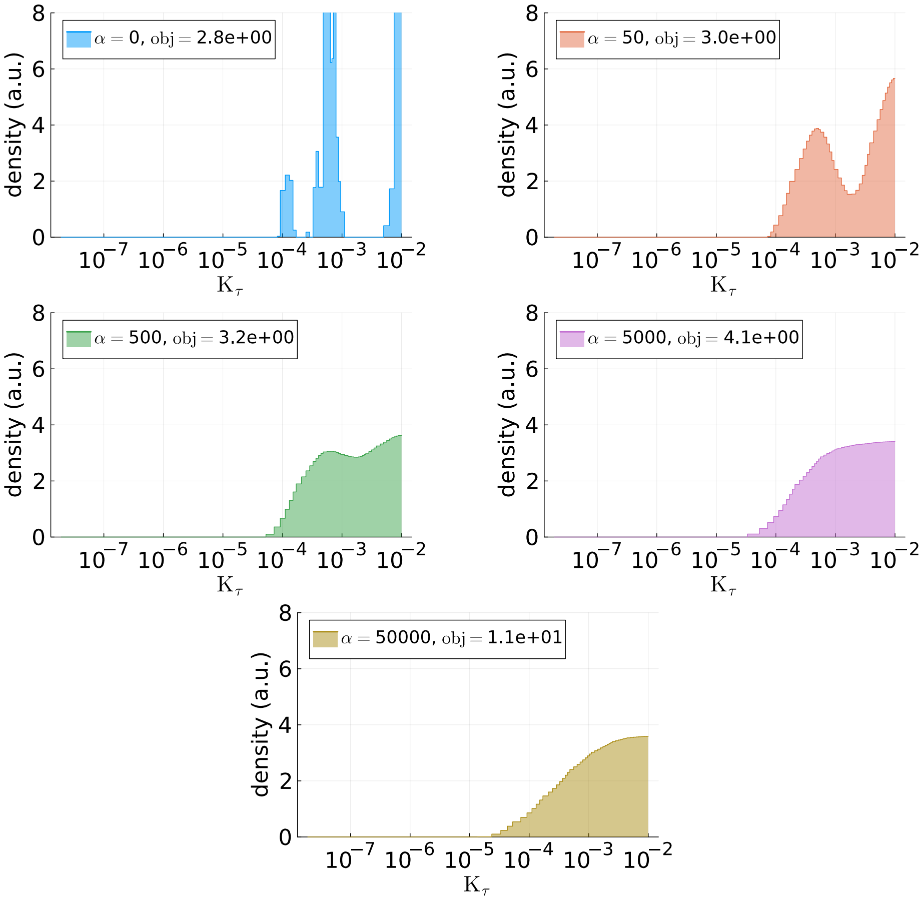


**Supplementary Figure 25**: Color-matched accessibility histograms for the different regularization parameters.

## 8.6 Histogram validation: Antibody mix

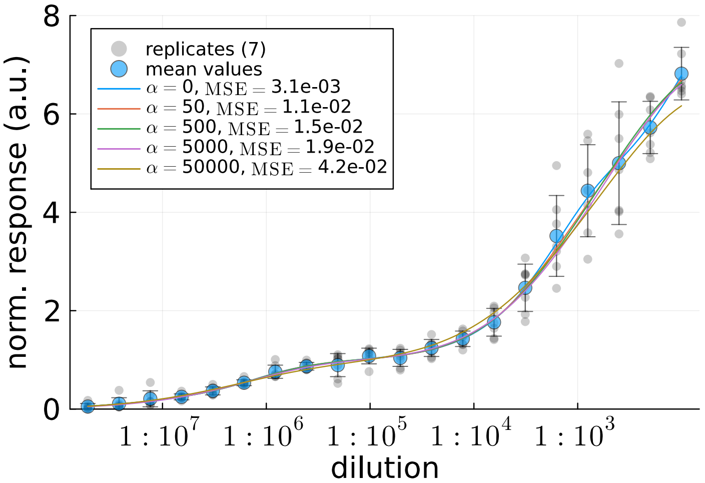


**Supplementary Figure 26**: Comparison of fitting results for different regularization parameters (mean values of $n = 7$ replicates; error bars = sample std).

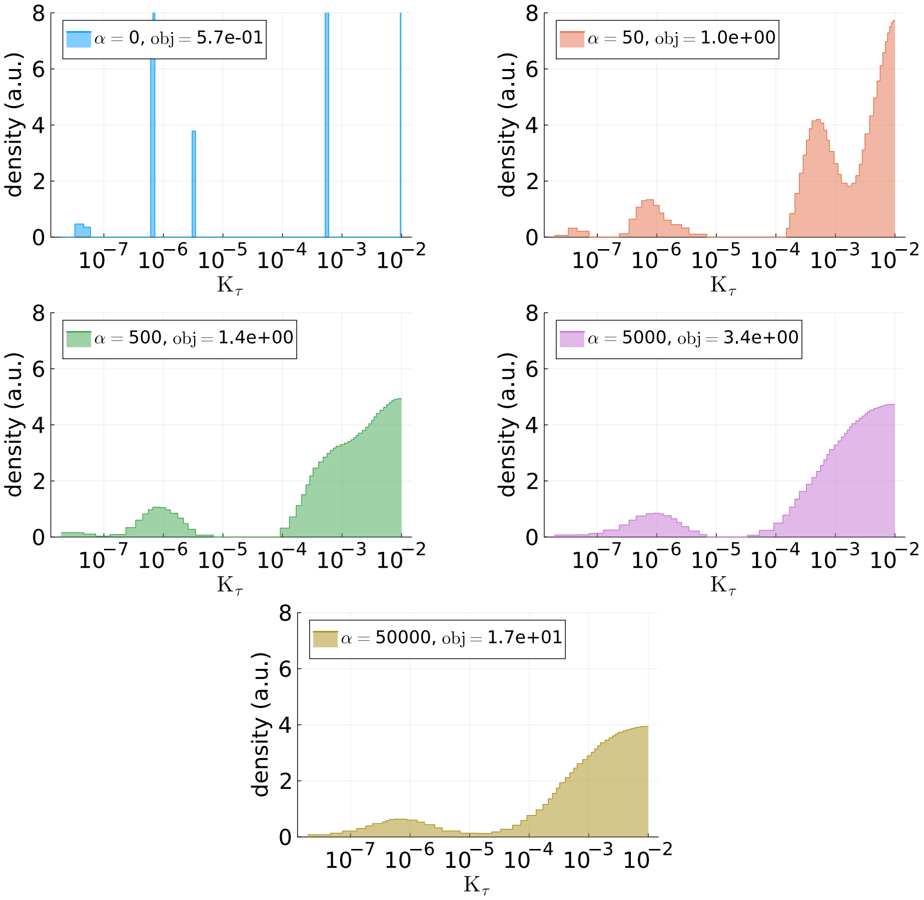


**Supplementary Figure 27**: Color-matched accessibility histograms for the different regularization parameters.

## 8.7 Anti-pRII antibody: Unstimulated HeLa cells

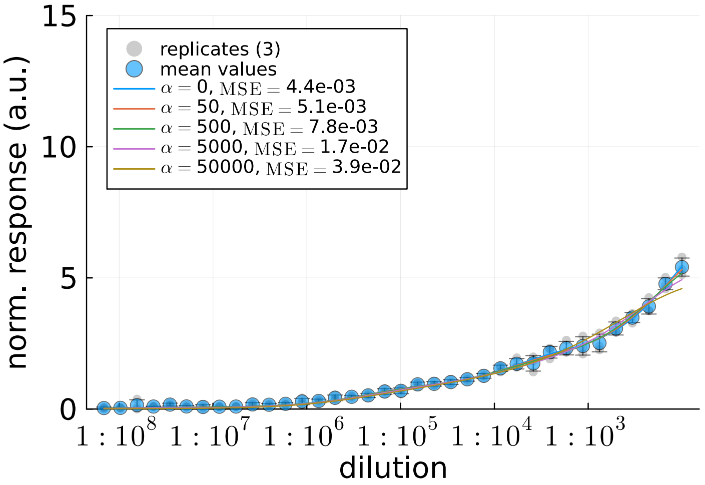


**Supplementary Figure 28**: Comparison of fitting results for different regularization parameters (mean values of $n = 3$ replicates; error bars = sample std).

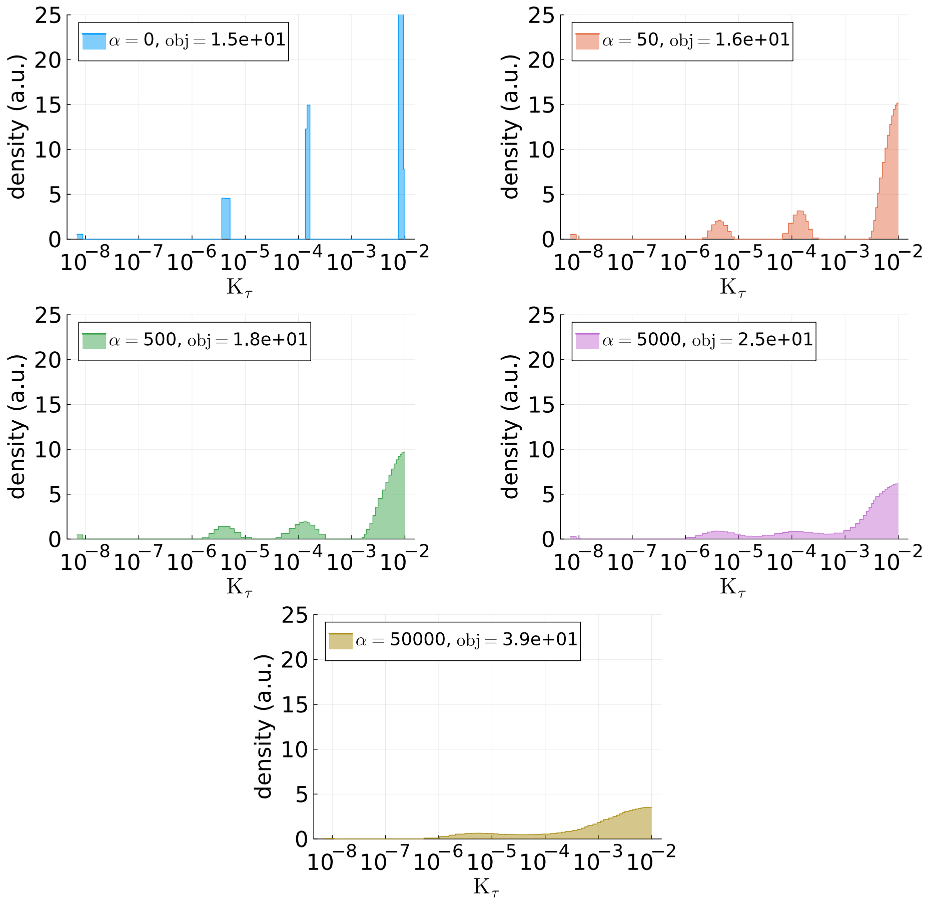


**Supplementary Figure 29**: Color-matched accessibility histograms for the different regularization parameters.

## 8.8 Anti-pRII antibody: cAMP-stimulated HeLa cells

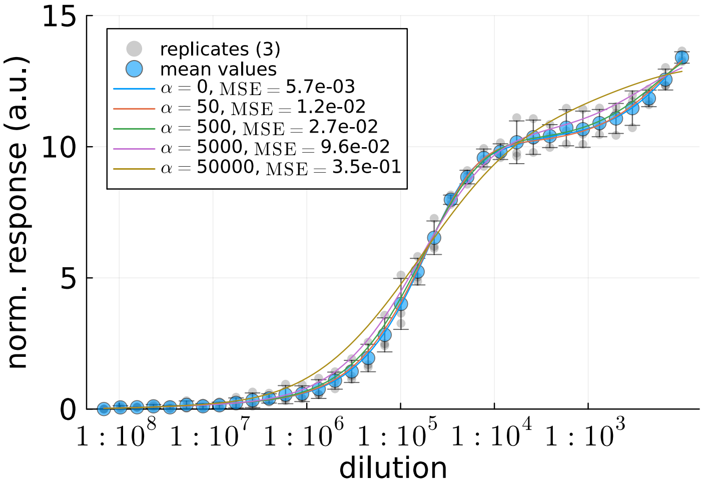


**Supplementary Figure 30**: Comparison of fitting results for different regularization parameters (mean values of $n = 3$ replicates; error bars = sample std, except for lowest dilution quotient, where error bars = $\pm 0.01$).

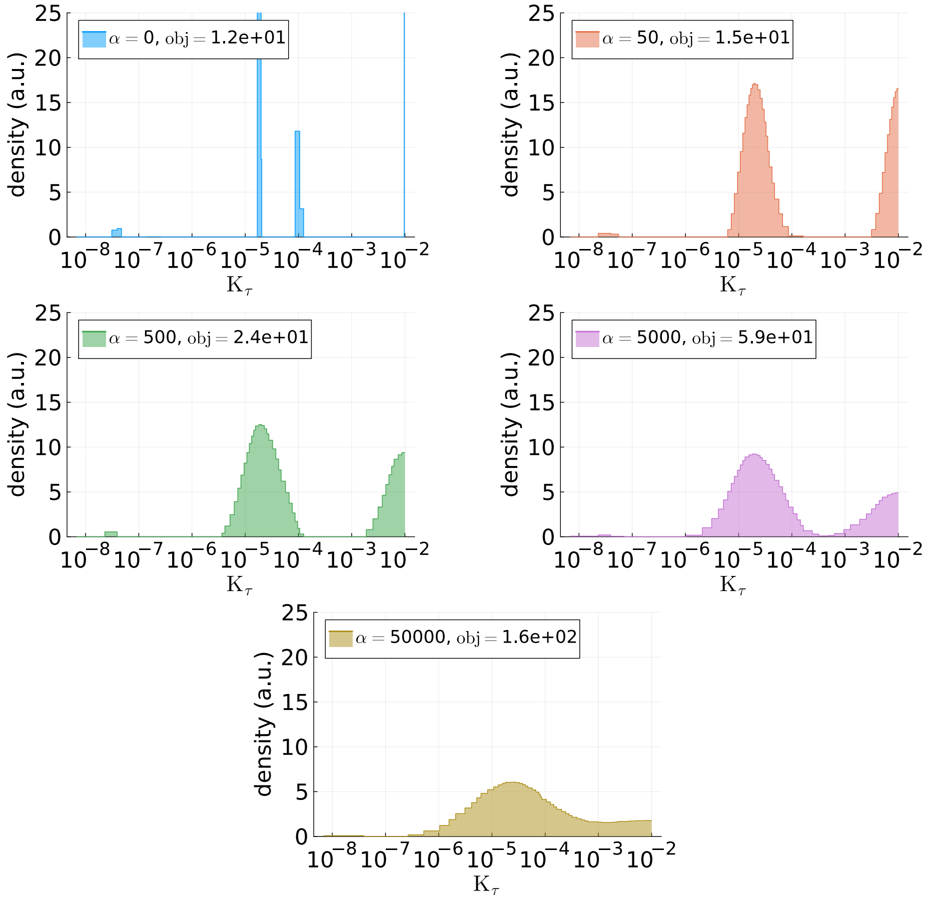


**Supplementary Figure 31**: Color-matched accessibility histograms for the different regularization parameters.

## 8.9 Anti-pRII antibody: Unstimulated DRG neurons with uniform errors

The measurement uncertainties are not the standard deviations of the replicates but $\pm 8$ for each data point (mean of replicates).

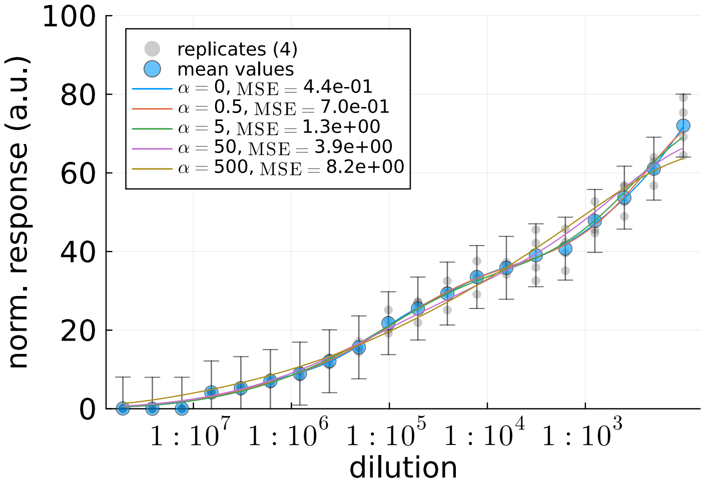


**Supplementary Figure 32**: Comparison of fitting results for different regularization parameters (mean values of $n = 4$ replicates; error bars $= \pm 8$).

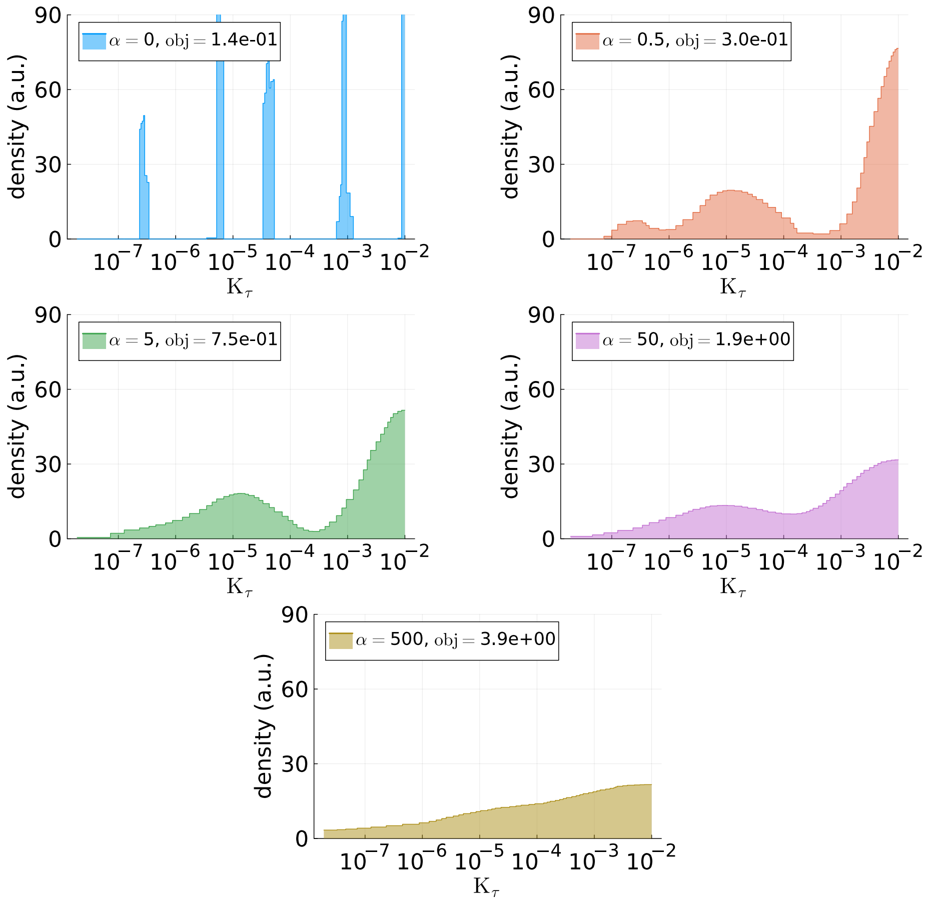


**Supplementary Figure 33**: Color-matched accessibility histograms for the different regularization parameters.

## 8.10 Anti-pRII antibody: cAMP-stimulated DRG neurons with uniform errors

The measurement uncertainties are not the standard deviations of the replicates but $\pm 8$ for each data point (mean of replicates).

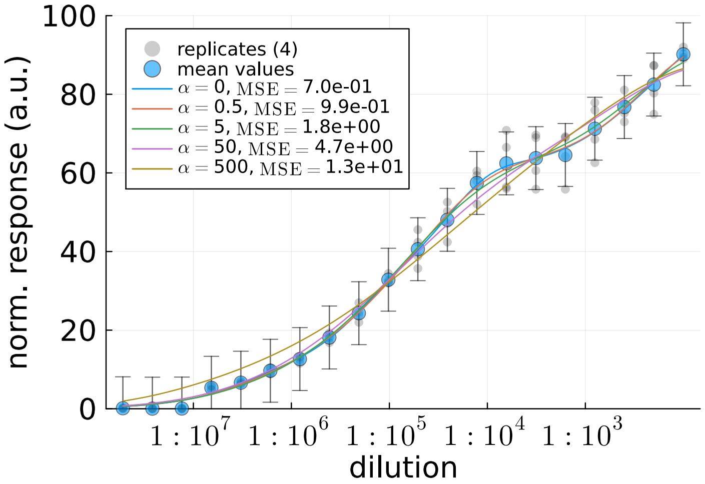


**Supplementary Figure 34**: Comparison of fitting results for different regularization parameters (mean values of $n = 4$ replicates; error bars $= \pm 8$).

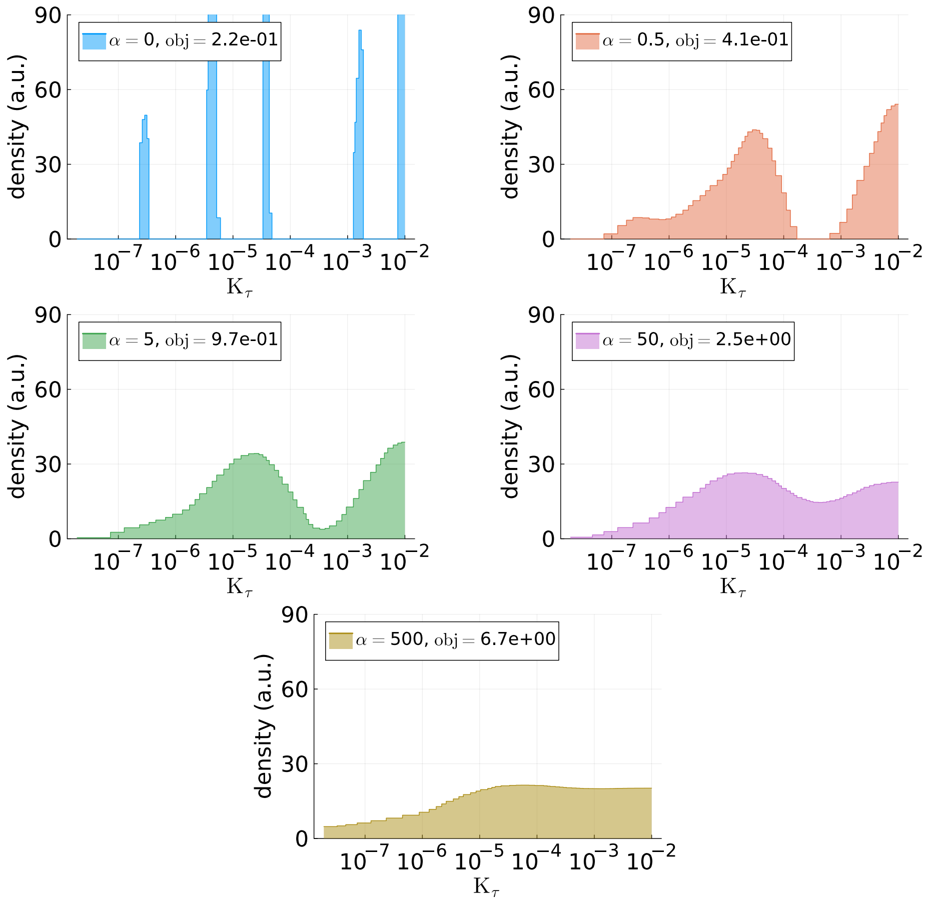


**Supplementary Figure 35**: Color-matched accessibility histograms for the different regularization parameters.

## 8.11 Anti-pRII antibody: Unstimulated DRG neurons

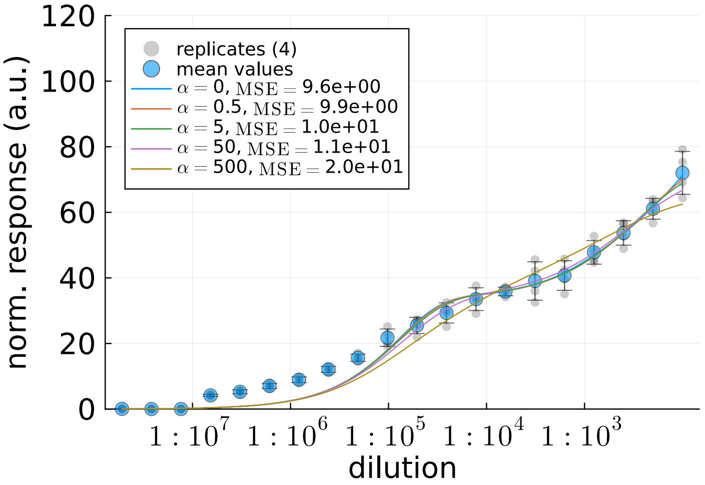


**Supplementary Figure 36**: Comparison of fitting results for different regularization parameters (mean values of $n = 4$ replicates; error bars $= \pm 8$).

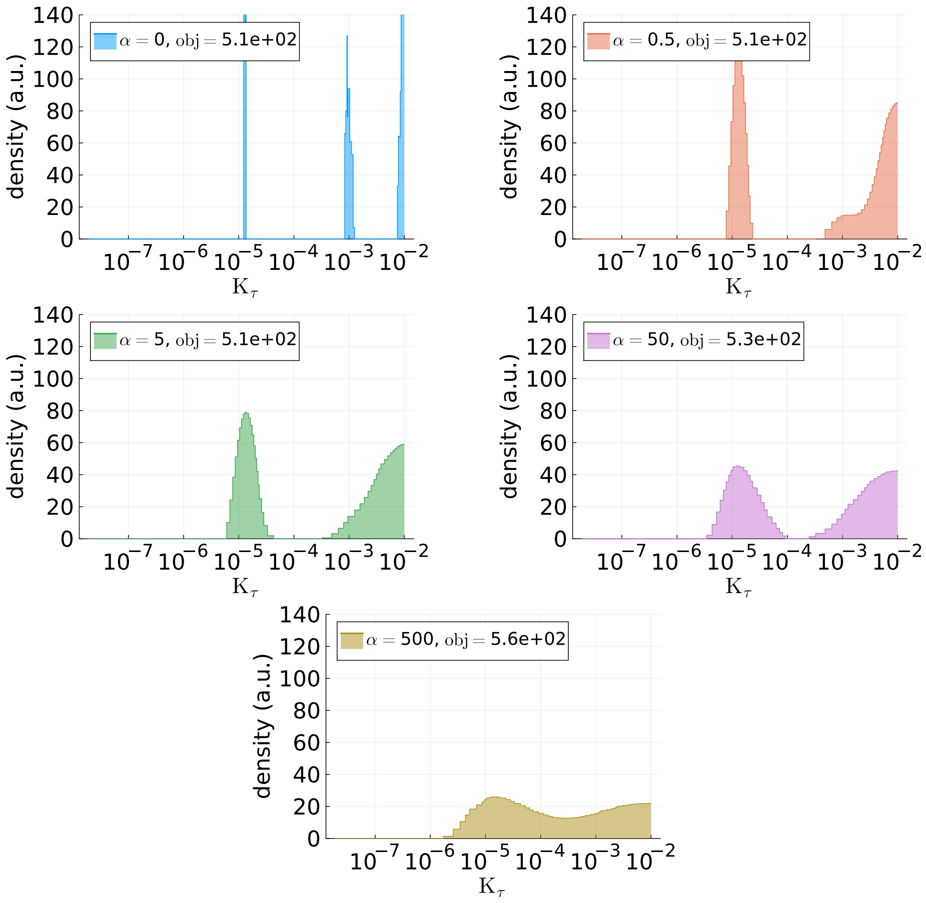


**Supplementary Figure 37**: Color-matched accessibility histograms for the different regularization parameters.

## 8.12 Anti-pRII antibody: cAMP-stimulated DRG neurons

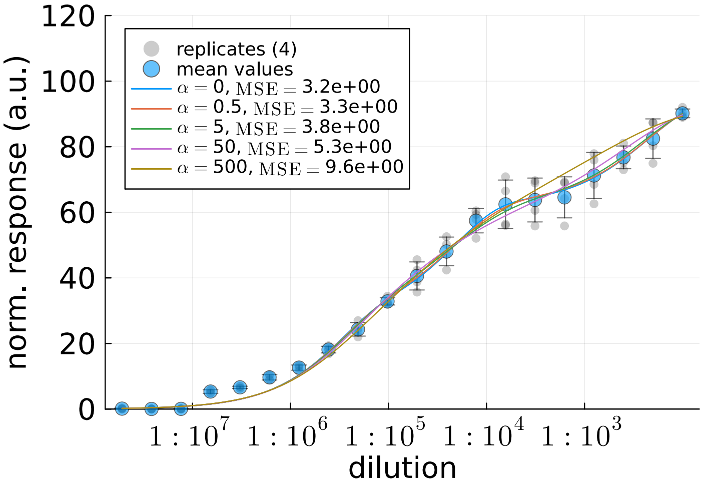


**Supplementary Figure 38**: Comparison of fitting results for different regularization parameters (mean values of $n = 4$ replicates; error bars $= \pm 8$).

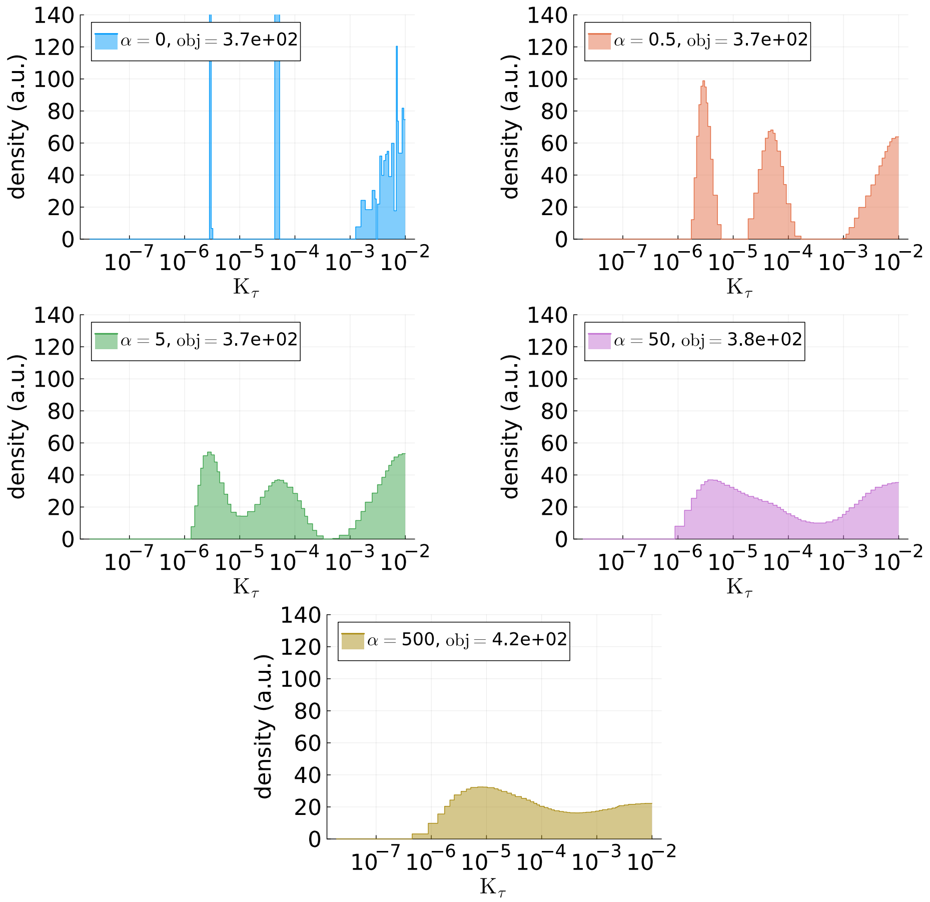


**Supplementary Figure 39**: Color-matched accessibility histograms for the different regularization parameters.

# 9 Removed replicates

Two replicates were removed from further analysis, one replicate for the anti-NF200 antibody and one replicate for the antibody mix. Figure 40a shows the dose-response data of the excluded anti-NF200 replicate (only NF200 signal) and Figure 40b shows the excluded antibody-mix replicate (anti-NF200 and anti-RPS11 signals). In both cases, it can be observed that the dose-response behavior (higher dose means higher response) is violated multiple times beyond the margin of noise for the anti-NF200 signal. Furthermore, the anti-RPS11 signal of the antibody mix condition contains an obvious outlier at the high-dilution-quotient end of the dose-response curve.

The violation of the dose-response behavior indicates that something must have gone wrong, since all other replicates follow the dose-response behavior within the margins of noise. What exactly went wrong is not known. However, since both faulty replicates are from the same plate, it can be speculated that some of the cells have dried

out during immunocytochemistry. Thus, we completely excluded the faulty replicates from all analyses.

**(a)** anti-NF200 condition **(b)** antibody-mix condition

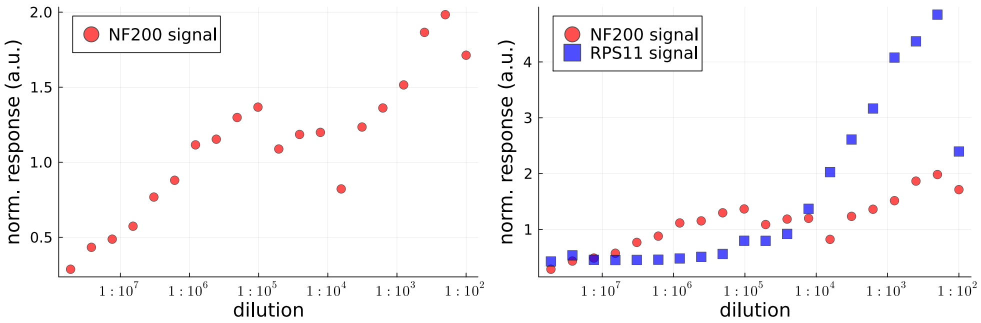


**Supplementary Figure 40**: Excluded replicates.

# References

[1] Weller, M.G.: Quality issues of research antibodies. Analytical Chemistry Insights **11**, 31614 (2016) https://doi.org/10.4137/aci.s31614

[2] Karlsson, R.: Biosensor binding data and its applicability to the determination of active concentration. Biophysical Reviews **8**(4), 347–358 (2016) https://doi.org/10.1007/s12551-016-0219-5

[3] Nayak, C., Rutenberg, A.: Quantification of fluorophore copy number from intrinsic fluctuations during fluorescence photobleaching. Biophysical Journal **101**(9), 2284–2293 (2011) https://doi.org/10.1016/j.bpj.2011.09.032

[4] Coffman, V.C., Wu, J.-Q.: Counting protein molecules using quantitative fluorescence microscopy. Trends in Biochemical Sciences **37**(11), 499–506 (2012) https://doi.org/10.1016/j.tibs.2012.08.002

[5] Bakker, E., Swain, P.S.: Estimating numbers of intracellular molecules through analysing fluctuations in photobleaching. Scientific Reports **9**(1) (2019) https://doi.org/10.1038/s41598-019-50921-7

[6] Edwards, P.R., Maule, C.H., Leatherbarrow, R.J., Winzor, D.J.: Second-order kinetic analysis of IAsys biosensor data: Its use and applicability. Analytical Biochemistry **263**(1), 1–12 (1998) https://doi.org/10.1006/abio.1998.2814
